# Meta analysis of ovulation induction effect and pregnancy outcome of acupuncture & moxibustion combined with clomiphene in patients with polycystic ovary syndrome

**DOI:** 10.3389/fendo.2023.1261016

**Published:** 2023-11-20

**Authors:** Lijie Yang, Wanqiu Yang, Minghan Sun, Linjie Luo, Hong Ru Li, Runqing Miao, Li Pang, Yajie Chen, Kun Zou

**Affiliations:** ^1^ Hospital of Chengdu University of Traditional Chinese Medicine, Center of Preventing and Treating Latent Disease, Chengdu, China; ^2^ Chengdu University of Traditional Chinese Medicine, College of Acupuncture and Tuina, Chengdu University of Traditional Chinese Medicine, Chengdu, China; ^3^ Sichuan Academy of Medical Sciences and Sichuan Provincial People’s Hospital, Central of Reproductive Medicine, Department of Obstetrics and Gynecology, Chengdu, China; ^4^ Wenjiang District People's Hospital of Chengdu, Intensive Care Unit, Chengdu, China; ^5^ West China Second University Hospital, Sichuan University, Chengdu, China

**Keywords:** PCOS, acupuncture & moxibustion, clomiphene, meta analysis ovulation rate, pregnancy outcome

## Abstract

**Objective:**

Using Mesh Meta Analysis to evaluate the efficacy of Acupuncture & Moxibustion, Clomiphene, Acupuncture & Moxibustion combined with Clomiphene for treating Polycystic Ovary Syndrome (PCOS), in order to provide evidence-based medical evidence for whether to recommend Acupuncture & Moxibustion or Combine western medicine to treat PCOS.

**Methods:**

Eight databases including The Cochrane Library, Pubmed, Embase, Web of Science, CNKI, Wanfang Date, VIP and CBM were searched by computer. The included research period is from the establishment of the database to May 2023, which concerned with randomized controlled trials involving Acupuncture & Moxibustion, Clomiphene, Acupuncture & Moxibustion combined with Clomiphene on ovulation induction and pregnancy outcome in patients with PCOS. The duration of the research paper is from 2016 to 2023.The inclusion criteria refer to the Rotterdam standards issued by the European Center for Human Reproduction and Embryology and the American Society of Reproductive Medicine in January 2003, or the Expert Consensus on the Diagnosis and Treatment of Polycystic Ovarian Syndrome by the Endocrinology Group of the Obstetrics and Gynecology Branch of the Chinese Medical Association. Simultaneously exclude related diseases, repetitive literature, as well as literature with incomplete abstract information and no original data provided. Two researchers independently screened the literature, extracted data, and evaluated the risk of bias included in the study, using Stata17.0 software for a mesh meta-analysis.

**Results:**

Six randomized controlled trials were included, covering 1410 PCOS patients. Three interventions included Acupuncture & Moxibustion, Clomiphene, Acupuncture & Moxibustion combined with Clomiphene. Mesh Meta Analysis showed that in terms of improving ovulation rate, there was no statistical difference between Acupuncture & Moxibustion (A), Clomiphene (B), Clomiphene combined with Acupuncture & Moxibustion (C) (P>0.05).Acupuncture & Moxibustion (A) versus Clomiphene (B) [MD=0.15,95% CI (-0.51,0.80)], Acupuncture & Moxibustion (A) versus Clomiphene combined with Acupuncture & Moxibustion (C) [MD=1.60,95% CI (0.97,2.23)], Clomiphene (B) versus Clomiphene combined with Acupuncture & Moxibustion (C) [MD=1.45,95% CI (0.91,1.99)]. In terms of pregnancy outcome, the difference between the three intervention methods was statistically significant (P<0.05). Acupuncture & Moxibustion (A) versus Clomiphene (B) [MD=-0.80,95% CI (-1.84,0.23)], Acupuncture & Moxibustion (A) versus Clomiphene combined with Acupuncture & Moxibustion (C) [MD=0.29,95% CI (-0.73,1.30)], and Clomiphene (B) versus Clomiphene combined with Acupuncture & Moxibustion (C) [MD=1.09,95% CI (0.39,1.79)], The order of pregnancy rate from high to low is Acupuncture & Moxibustion combined with Clomiphene (C), Acupuncture & Moxibustion (A), Clomiphene (C).In terms of influencing endometrial thickness, the difference between the three intervention methods was statistically significant (P<0.05). Acupuncture & Moxibustion (A) versus Clomiphene (B) [MD=-0.84,95% CI (-1.87,0.19)], Acupuncture & Moxibustion (A) versus Acupuncture & Moxibustion combined with Clomiphene (C) [MD=0.26,95% CI (-1.01,1.53)], Clomiphene (B) versus Acupuncture & Moxibustion combined with Clomiphene (C) [MD=1.10,95% CI (0.36,1.84)], Acupuncture & Moxibustion combined with Clomiphene (C) has the best effect on improving endometrial thickness. In subgroup analysis, the effect of Acupuncture & Moxibustion treatment frequency on ovulation rate and pregnancy rate was not statistically significant. The combination of Acupuncture & Moxibustion, Electroacupuncture and warm Acupuncture & Moxibustion has no effect on the pregnancy rate, but the combination of Electroacupuncture and Clomiphene has the best effect on improving the ovulation rate. In the observation of adverse reactions, compared with clomiphene alone, Acupuncture & Moxibustion combined with Clomiphene can reduce the occurrence of Luteinized Unruptured Follicle Syndrome (LUFS) and Ovarian Hyperstimulation Syndrome (OHSS), and reduce the occurrence of physical adverse reactions such as nausea, vomiting, headache and dermatitis.

**Conclusion:**

Acupuncture & Moxibustion is effective in improving the ovulation promoting effect and pregnancy outcome of PCOS patients. The ovulation promoting effect of Acupuncture & Moxibustion or combined with Clomiphene is similar to that of Clomiphene alone, but Acupuncture & Moxibustion combined with Clomiphene has more advantages in improving the pregnancy rate of PCOS, and it also can reduce the adverse reactions of Clomiphene alone. Acupuncture & Moxibustion can be used as a recommended treatment for PCOS. More cases should also be included in the subgroup analysis to study the impact of Acupuncture & Moxibustion programs on clinical efficacy and further optimize the Acupuncture & Moxibustion treatment program.

**Systematic review registration:**

https://www.crd.york.ac.uk/PROSPERO/#myprospero, identifier (CRD42023433057).

## 1 Introduction

PCOS is a common endocrine and metabolic disorder in women of childbearing age, and is the main cause of anovulatory infertility (1). Promoting follicular development is a key link in improving the outcome of anovulatory infertility in PCOS. Clomiphene, a first-line ovulation promoting drug in clinical practice, has a high ovulation rate but poor implantation effect (2, 3). During ovulation promotion, it can also induce the occurrence of ovarian hyperstimulation syndrome or luteinized unruptured follicle syndrome (4, 5).

Acupuncture & Moxibustion, as a non drug treatment method, has simple operation, high acceptance (6), and few adverse reactions. It has attracted much attention in the reproductive field in recent years. Many studies have confirmed that Acupuncture & Moxibustion plays a positive role in promoting follicular development (7, 8), improving pregnancy outcomes (9, 10) and other links, and can make up for the lack of clinical treatment of Clomiphene (11–14). However, studies have reported that Acupuncture & Moxibustion has no effect on the improvement of PCOS live birth rate (15), and there is no systematic evaluation of the controversial effect. Therefore, a Mesh Meta Analysis is used to compare the ovulation rate, pregnancy outcome The influence of sex hormone level provides evidence-based medical evidence for whether to recommend Acupuncture & Moxibustion or combine western medicine to treat PCOS.

## 2 Materials and methods

### 2.1 Retrieval strategy

Computer search of The Cochrane Library, Pubmed, Embase, Web of Science, China National Knowledge Infrastructure (CNKI), Wanfang Date, VIP, and China Biomedical Literature Database (CBM), with a search deadline of May 2023. The search term is a combination of subject words and free words. The English search formula is (Polycyclic Ovary Syndrome OR PCOS) AND (Clomiphene), (Polycyclic Ovary Syndrome OR PCOS) AND (Acquire). The Chinese search formula is [(clomiphene) or (clomiphene)] and [(polycystic ovary syndrome)], [Acupuncture & Moxibustion] and [(polycystic ovary syndrome)], [Moxibustion] and [(polycystic ovary syndrome)], [Electroacupuncture] and [(polycystic ovary syndrome)].

### 2.2 Criteria for inclusion and exclusion of literature

#### 2.2.1 Research type

publicly published clinical Randomized controlled trial (RCT), and the language is limited to English or Chinese.

#### 2.2.2 Subjects

PCOS patients diagnosed according to the Rotterdam Standard (16) issued by the European Research Center for Human Reproduction and Embryology and the American Society of Reproductive Medicine in January 2003, or the Expert Consensus on Diagnosis and Treatment of Polycystic Ovary Syndrome (17, 18) issued by the Endocrinology Group of the Gynecology and Obstetrics Branch of the Chinese Medical Association in 2008 and 2018, and the age ≥ 18 years old.

#### 2.2.3 Intervention measures

Clomiphene plus Acupuncture & Moxibustion or Acupuncture & Moxibustion alone.

#### 2.2.4 Control measures

Clomiphene, sham Acupuncture, or placebo.

#### 2.2.5 Outcome indicators

The main outcome indicators are pregnancy rate and ovulation rate; The secondary outcome measure is the regulatory effect of the intervention plan on follicular development and endometrium.

#### 2.2.6 Subgroup analysis:

The influence of different Acupuncture & Moxibustion frequency on clinical efficacy, and the influence of different Acupuncture & Moxibustion methods on clinical efficacy.

#### 2.2.7 Exclusion criteria

① Research on other diseases such as hyperprolactinemia, thyroid dysfunction, premature ovarian failure, androgen secreting adrenal gland, ovarian tumor, hyperandrogenism caused by pituitary or hypothalamic amenorrhea, Menstrual disorder and ovulation disorders.

② Non RCT, including review literature, animal and *in vitro* trial literature, conference literature, meta-analysis literature, retrospective analysis literature, clinical case reports, etc.

③ The sample size included in the study is less than 20 cases.

④ The experimental group was treated with traditional Chinese medicine injection, oral Chinese medicine, and topical Chinese medicine.

⑤ The patient has medication history in these patients like anti-diabetic drug or statin or any hormonal treatment.

⑥ Repeated publications.

⑦ Literature with incomplete abstract information and no original data provided.

### 2.3 Literature screening and data extraction

Two researchers independently conducted literature screening, data extraction, and cross checking. Differences of opinion were reached through discussion or decided by the third senior researcher. The initial screening usually uses reading questions and abstracts, and the re screening reads the entire text.

The data extraction adopts a unified data extraction table, which includes baseline characteristics of the study subjects such as title, author, sample size, average age, gender, disease course, intervention measures (type, course of treatment), outcome indicators, etc.

### 2.4 Risk assessment of bias included in the study

The Cochrane RoB tool was used to evaluate the bias risk of inclusion in the study, and R.4.30 software was used for chart output.

### 2.5 Statistical analysis

Using the relevant installation packages and commands in Stata17.0, the data was processed and a network relationship diagram, meta-analysis diagram (prediction interval diagram), validity order diagram, funnel diagram, etc. were drawn. A network meta-analysis was conducted on the main indicators of ovulation rate, pregnancy rate, secondary indicators of follicle size, and endometrial thickness.

## 3 Results

### 3.1 Document retrieval results

**Figure 1:**
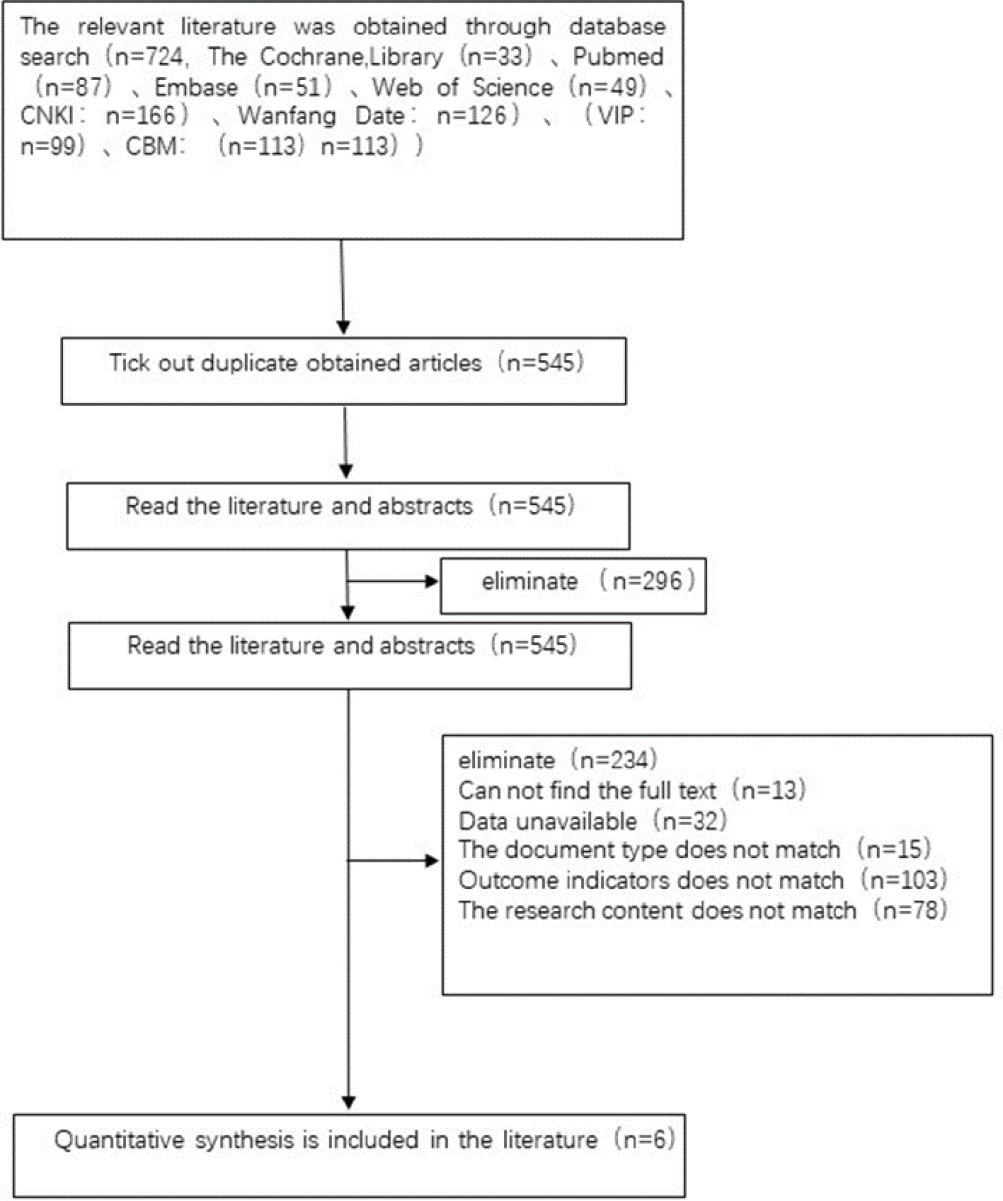
Literature screening process and results.

A preliminary search of 724 articles was conducted, and the databases searched and the number of detected articles were as follows: The Cochrane, Library (n=33), Pubmed (n=87), Embase (n=51), Web of Science (n=49), CNKI: n=166), Wanfang Date: n=126), (VIP: n=99), CBM: (n=113). After layer-by-layer screening, literature that did not meet diagnostic criteria, was repeatedly published, the abstract information was incomplete, and no original data was provided was removed. Finally, 6 articles (5, 11–15) were included, with a total of 1410 patients. The literature screening process and results are detailed in **Figure 1**.

The study was carried out in China, the research period was from June 2012 to December 2020, with three different interventions, including Acupuncture & Moxibustion or Acupuncture & Moxibustion combined with placebo (300 cases), clomiphene (231 cases), Acupuncture & Moxibustion combined with clomiphene (371 cases), sham Acupuncture & Moxibustion combined with clomiphene (250 cases), and sham Acupuncture & Moxibustion combined with placebo (250 cases). The research observation content includes the main outcome indicators of ovulation rate (study 1402 cases) and pregnancy outcome (study 1402 cases), secondary outcome indicators of follicular development (study 202 cases) and endometrial development (study 300 cases), and adverse reactions (study 1200 cases).

Reasons for eliminate 296 articles:

① Not included in the inclusion criteria for diseases.

② Non RCT.

③ The sample size included in the study is less than 20 cases.

④ The experimental group was treated with traditional Chinese medicine injection, oral Chinese medicine, and topical Chinese medicine.

⑤ Repeated publications.

⑥ Literature with incomplete abstract information and no original data provided.

### 3.2 Basic characteristics of inclusion in the study

Among the six studies included, one was in English and five were in English. The basic characteristics of the included studies are shown in **Supplementary Table 1**. Among the inclusion criteria, four studies (11–13, 15) referred to the 2003 Rotterdam standards, and two studies (5, 14) referred to the “Expert Consensus on the Diagnosis and Treatment of Polycystic Ovarian Syndrome” by the Endocrinology Group of the Obstetrics and Gynecology Branch of the Chinese Medical Association. In the comparison of intervention methods, the intervention methods of two studies (12, 14) were Acupuncture & Moxibustion plus clomiphene and clomiphene, the intervention methods of one study (13) were warm Acupuncture & Moxibustion plus clomiphene and clomiphene, the intervention methods of one study (5) were Acupuncture & Moxibustion plus clomiphene, the intervention methods of one study (11) were Electroacupuncture plus clomiphene and clomiphene, and the intervention methods of one study (15) were electroacupuncture plus clomiphene Comparison of electroacupuncture combined with placebo, sham Acupuncture & Moxibustion combined with clomiphene and sham Acupuncture & Moxibustion combined with placebo. In the observation of outcome indicators, all 6 studies observed pregnancy rate and ovulation rate. In the observation of secondary outcome indicators, three studies (5, 11, 12) observed the effect of intervention methods on endometrial thickness, one study (12) observed the effect of intervention methods on follicle diameter, and one study (13) observed the effect of intervention methods on follicle number. In the observation of adverse reactions, three studies (11, 12, 15) reported adverse reactions.

### 3.3 Data quality evaluation

**Figure 2:**
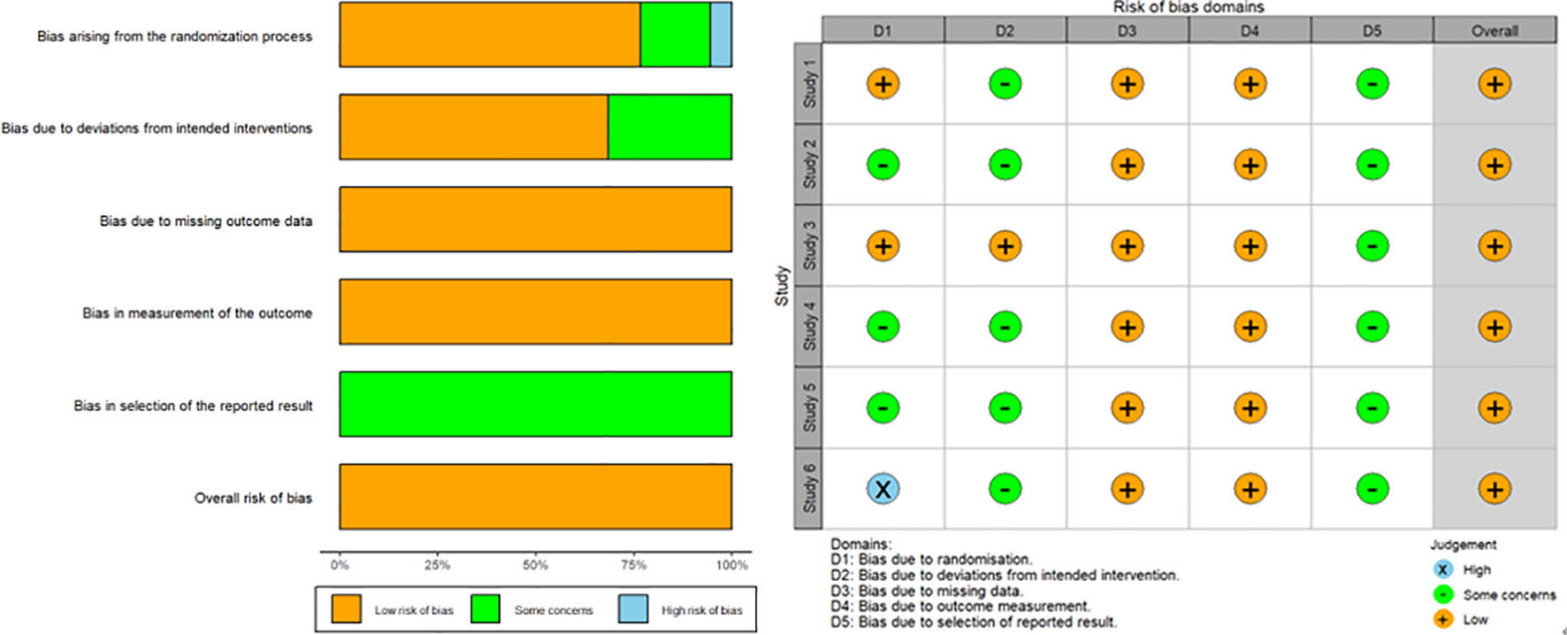
Data quality evaluation ROB.

In the random allocation method, three studies (5, 11, 14) mainly used the random number table method for random allocation, one study (15) used an interactive online computer program, and two studies (12, 13) used a simple random method. Conduct literature quality evaluation on the included studies using the Cochrane scale, and output charts using R.4.30 software. The specific results are shown in **Figure 2**. Except for the selection bias in study 6 (14), which is in a high-risk state, all other studies are in a relatively low risk state. As for all included studies, it can be considered that this network meta-analysis includes no high risk and can draw relatively reliable conclusions.

### 3.4 Analysis results of main indicators

#### 3.4.1 Analysis and comparison of network relationships

##### 3.4.1.1 Ovulation rate

**Figure 3:**
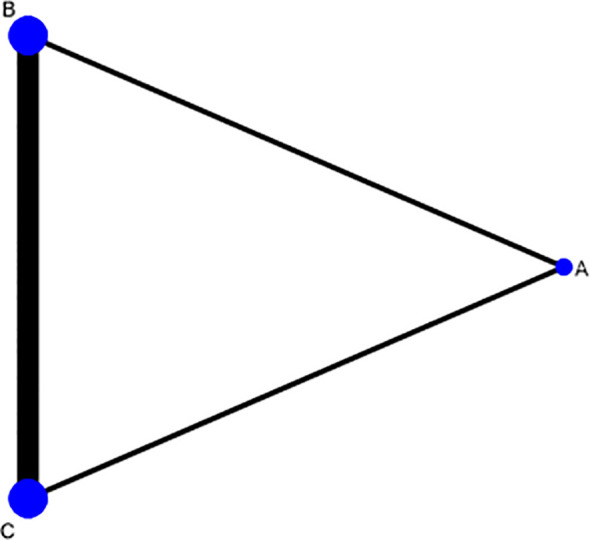
Comparison of ovulation rate network relationship analysis among three intervention measures.

Three intervention measures were Acupuncture & Moxibustion (A), Clomiphene (B), and Acupuncture combined with Clomiphene (C). The network diagram shows that the comparison between Clomiphene plus Acupuncture & Moxibustion (C) and Clomiphene (B) is the most popular, the comparison between Acupuncture & Moxibustion (A) and Clomiphene (B) is more popular, and the comparison between Acupuncture & Moxibustion combined with Clomiphene (C) and Acupuncture & Moxibustion (A) is the least popular. The comparison between the three intervention measures forms a closed loop, indicating both direct and indirect comparisons (**Figure 3**).

##### 3.4.1.2 Pregnancy rate

**Figure 4:**
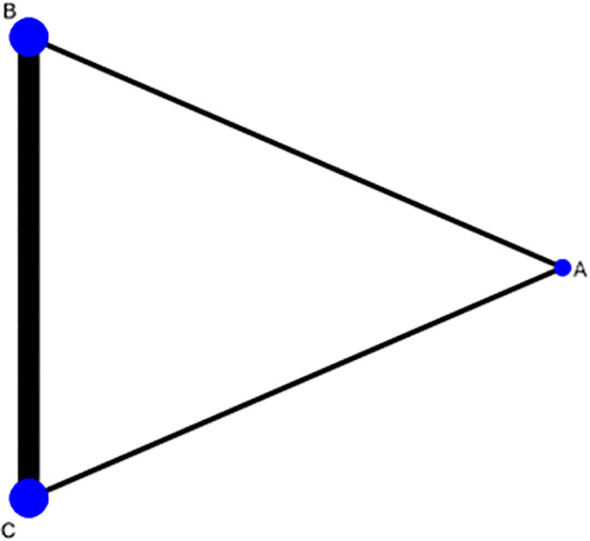
Network diagram of pregnancy rate comparison among three intervention measures.

Three intervention measures were Acupuncture & Moxibustion (A), Clomiphene (B), and Acupuncture combined with Clomiphene (C). The network diagram shows that the comparison between clomiphene plus Acupuncture & Moxibustion (C) and clomiphene (B) is the most popular, the comparison between Acupuncture & Moxibustion (A) and clomiphene (B) is more popular, and the comparison between clomiphene plus Acupuncture & Moxibustion (C) and Acupuncture & Moxibustion (A) is the least popular. The comparison between the three intervention measures forms a closed loop, indicating both direct and indirect comparisons (**Figure 4**).

#### 3.4.2 Bias risk assessment

##### 3.4.2.1 Ovulation rate

**Figure 5:**
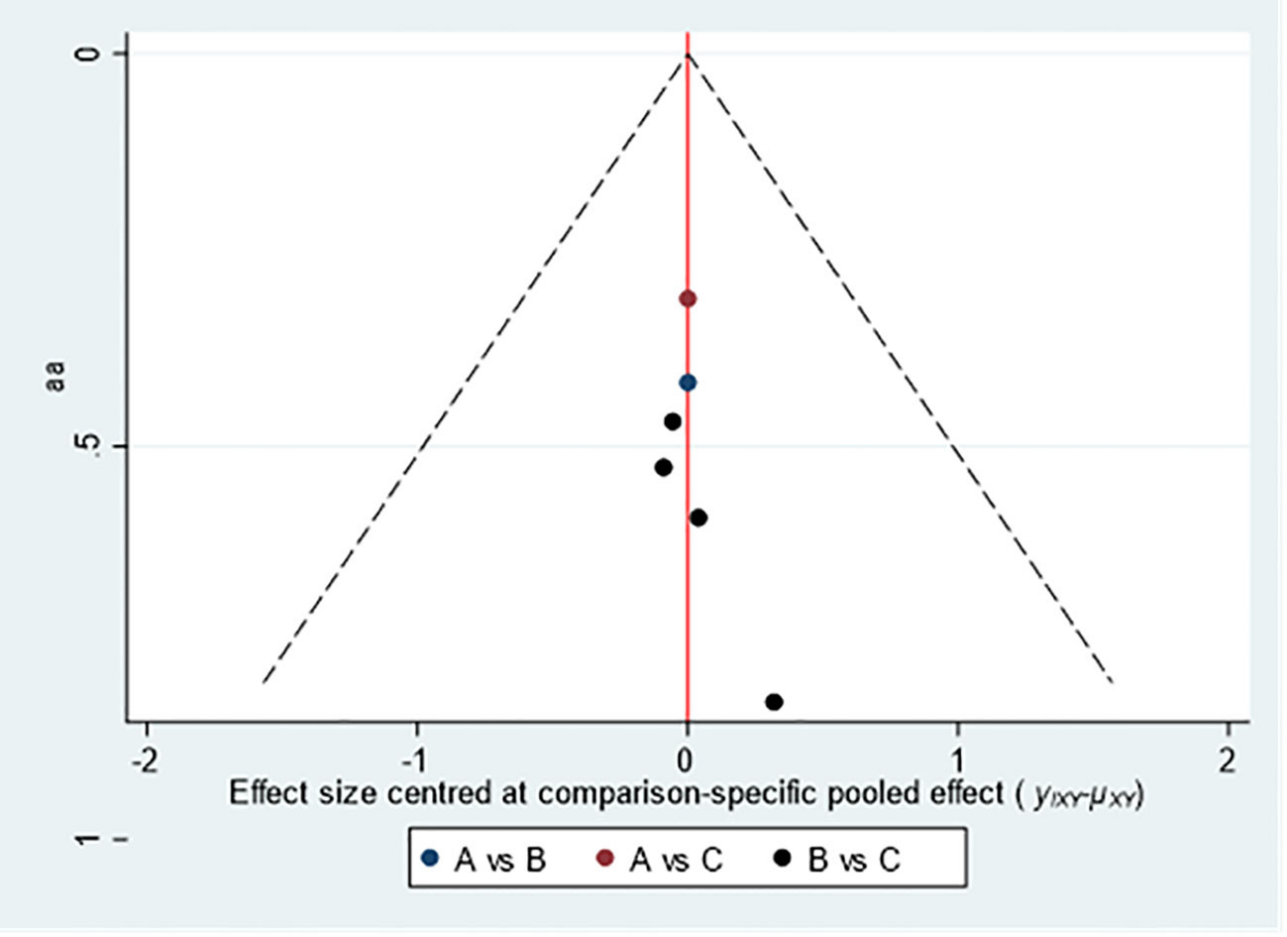
Funnel Chart comparison of ovulation rates among three intervention measures.

The three intervention measures included Acupuncture & Moxibustion (A), Clomiphene (B), and Acupuncture & Moxibustion plus clomiphene (C). The funnel plot shows that the literature included in the three intervention measures is relatively evenly distributed on both sides of the central axis, and the consistency and bias of all literature data included are significant (**Figure 5**).

##### 3.4.2.2 Comparison of pregnancy rates

**Figure 6:**
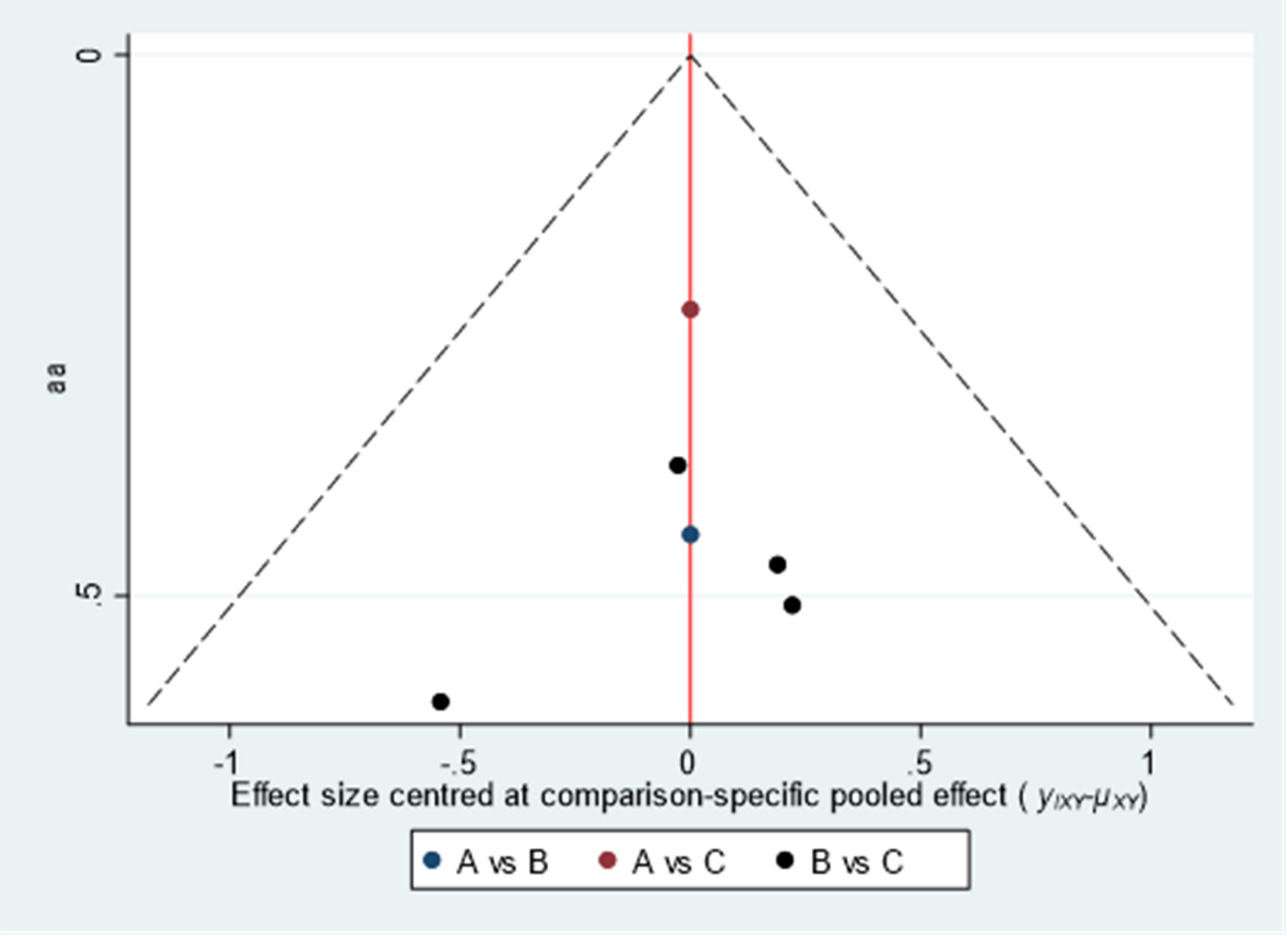
Funnel Chart comparison of pregnancy rates among three intervention measures.

The three intervention measures included Acupuncture & Moxibustion (A), Clomiphene (B), and Acupuncture & Moxibustion plus clomiphene (C). The funnel plot shows that the literature included in the three intervention measures is relatively evenly distributed on both sides of the central axis, and the consistency and bias of all literature data included are significant (**Figure 6**).

#### 3.4.3 Mesh meta analysis results

##### 3.4.3.1 Ovulation rate

**Figure 7:**
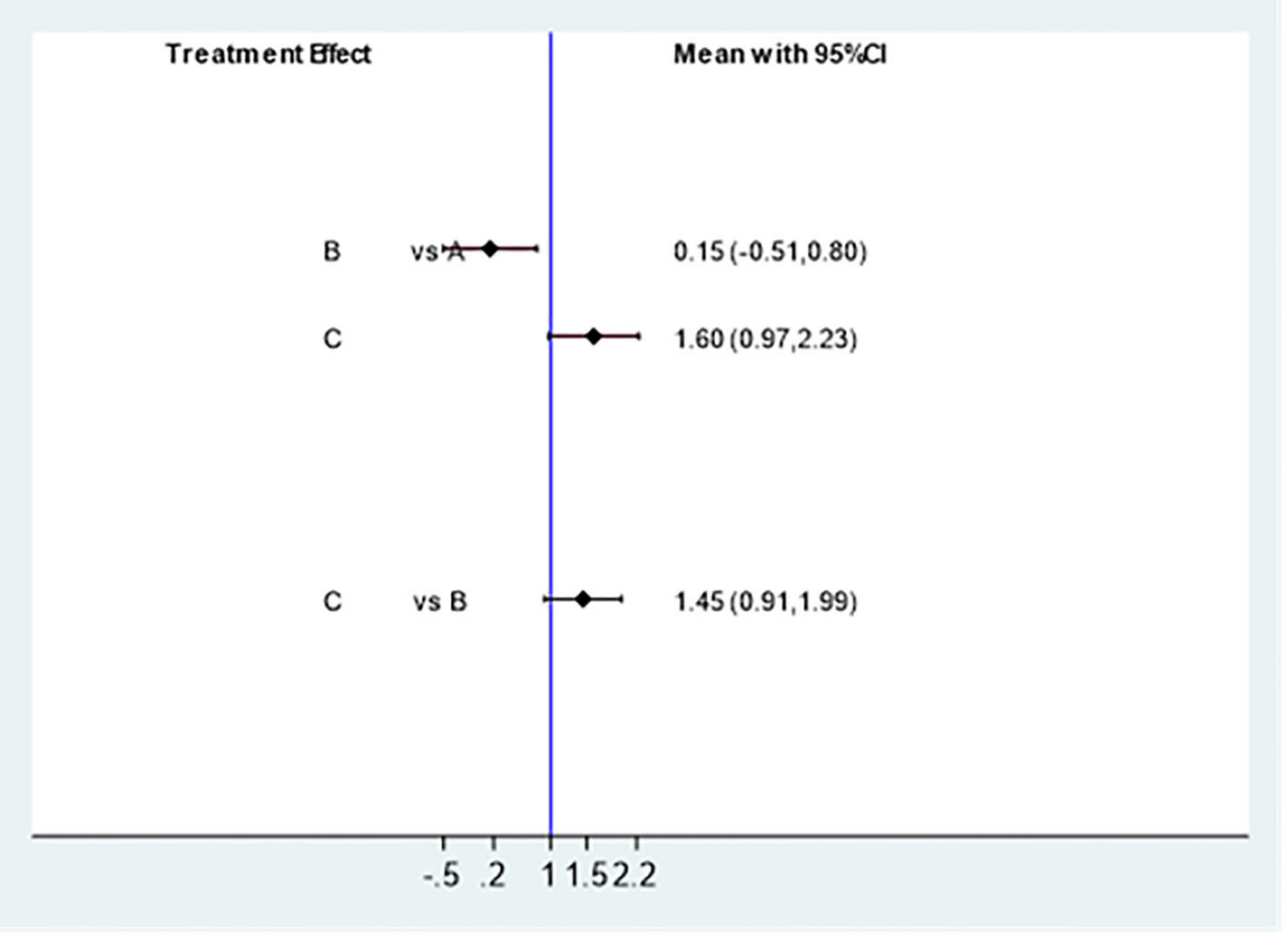
Comparison of statistical differences in ovulation rate network analysis among three intervention methods.

Effectively included in 6 studies (5, 11–15) to analyze the impact of different intervention methods on ovulation rate. Three intervention methods: 273 cases were studied with Acupuncture & Moxibustion (A), 178 cases were studied with clomiphene (B), and 464 cases were studied with Acupuncture & Moxibustion and clomiphene (C). The reticular meta-analysis results showed that Acupuncture & Moxibustion (A) was compared with clomiphene (B) [MD=0.15,95% CI (-0.51,0.80)], Acupuncture & Moxibustion (A) was compared with clomiphene combined with Acupuncture & Moxibustion (C) [MD=1.60,95% CI (0.97,2.23)], and clomiphene (B) was compared with Acupuncture & Moxibustion combined with clomiphene (C) [MD=1.45,95% CI (0.91,1.99)], P: 0.0836, P>0.05. There was no statistically significant difference in the comparison between the appeal groups (**Figure 7**).

##### 3.4.3.2 Pregnancy rate

**Figure 8:**
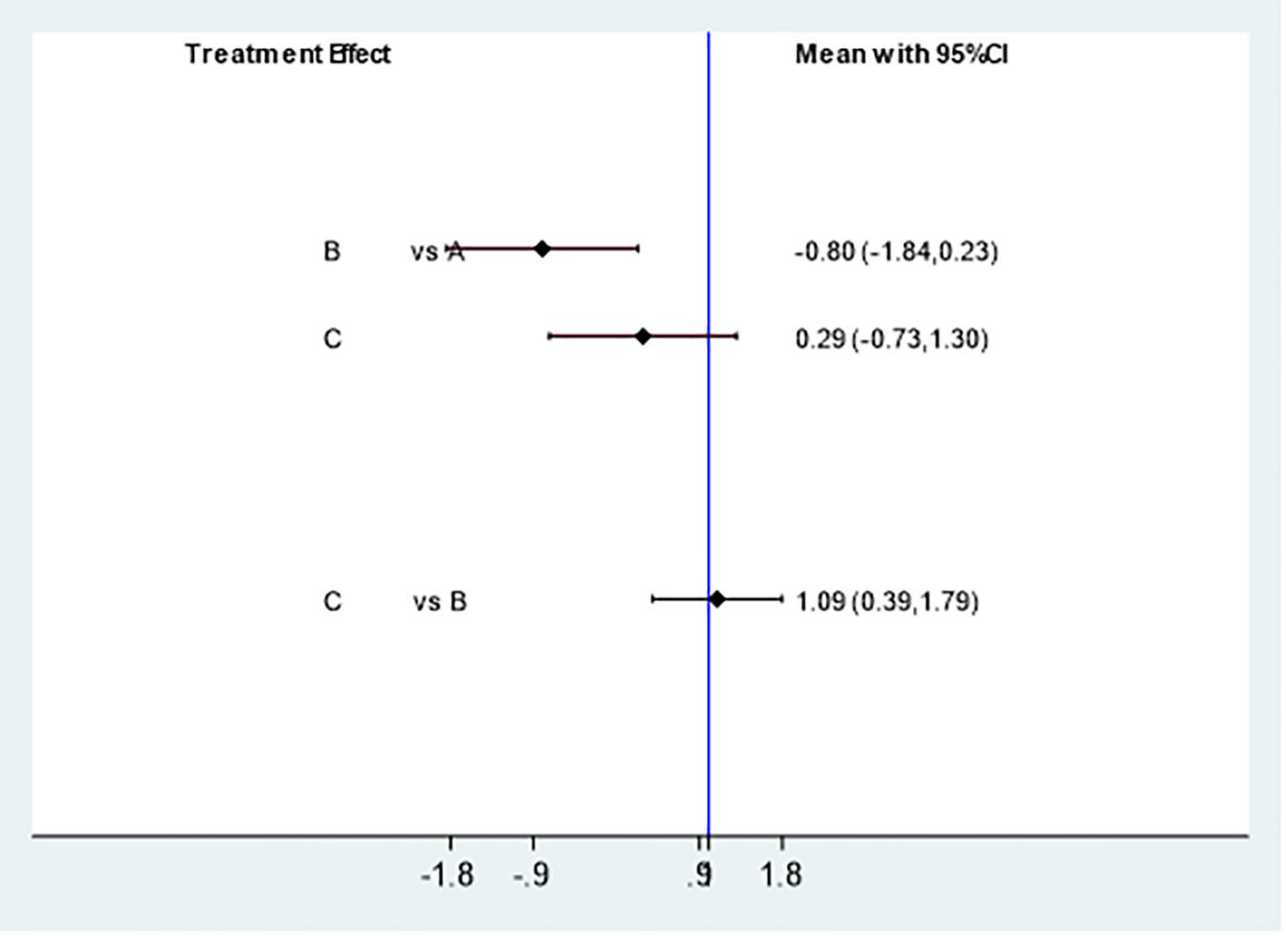
Comparison of statistical differences in pregnancy rate network analysis among three intervention methods.

Effectively included in 6 studies (5, 11–15) to analyze the impact of different intervention methods on pregnancy rate. Three intervention methods: 273 cases were studied with Acupuncture & Moxibustion (A), 178 cases were studied with Clomiphene (B), and 464 cases were studied with Acupuncture & Moxibustion and Clomiphene (C). The reticular meta-analysis results showed that Acupuncture & Moxibustion (A) was compared with Clomiphene (B) [MD=-0.80,95% CI (-1.84,0.23)], Acupuncture & Moxibustion (A) was compared with Acupuncture & Moxibustion combined with Clomiphene (C) [MD=0.29,95% CI (-0.73,1.30)], and Clomiphene (B) was compared with Acupuncture & Moxibustion combined with Clomiphene (C) [MD=1.09,95% CI (0.39,1.79)], P=0.0009, P<0.05. There is a statistical difference in the comparison between the appeal groups (**Figure 8**).

#### 3.4.4 Efficacy ranking analysis results

##### 3.4.4.1 Ovulation rate

Using SUCRA to analyze the difference in the efficacy of three interventions to promote ovulation in PCOS patients, the results showed that the ovulation rate from high to low was Acupuncture & Moxibustion combined with Clomiphene (C), Acupuncture & Moxibustion (A), Clomiphene (B) (**Supplementary Figure 1**).

##### 3.4.4.2 Pregnancy rate

Using SUCRA to analyze the difference in the efficacy of three interventions to improve the pregnancy outcome of PCOS patients, the results showed that the order of pregnancy rate from high to low was Acupuncture & Moxibustion combined with Clomiphene (C), Acupuncture & Moxibustion (A), Clomiphene B (**Supplementary Figure 2**).

### 3.5 Analysis of secondary indicators

#### 3.5.1 Mesh meta analysis results

##### 3.5.1.1 Endometrial thickness

**Figure 9:**
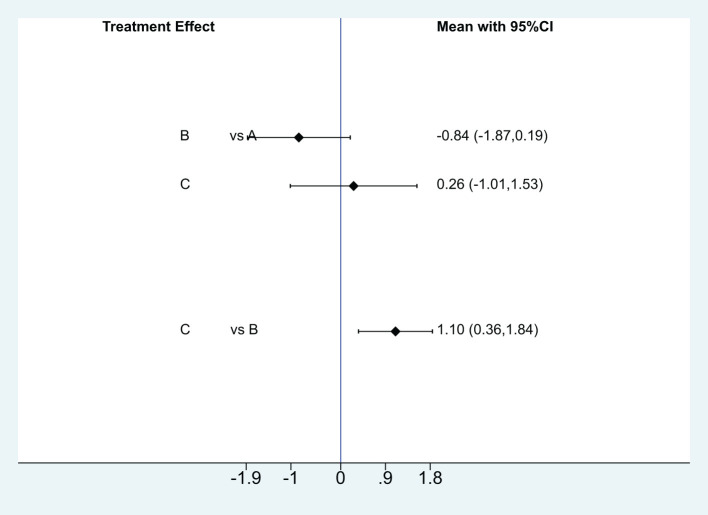
Network Meta Analysis Results of the effects of three intervention methods on endometrial thickness.

Four articles (5, 13–15) were effectively included in the analysis to analyze the impact of different intervention methods on endometrial thickness. Three intervention methods: 50 cases were studied with Acupuncture & Moxibustion (A), 97 cases were studied with Clomiphene (B), and 98 cases were studied with Acupuncture & Moxibustion combined with Clomiphene (C). The reticular meta-analysis results showed that Acupuncture & Moxibustion (A) was compared with Clomiphene (B) [MD=-0.84,95% CI (-1.87,0.19)], Acupuncture & Moxibustion (A) was compared with Acupuncture & Moxibustion combined with Clomiphene (C) [MD=0.26,95% CI (-1.01,1.53)], clomiphene (B) was compared with Acupuncture & Moxibustion combined with Clomiphene (C) [MD=1.10,95% CI (0.36,1.84)], P=0.0037, P<0.05, and the difference was statistically significant (See **Figure 9**).

#### 3.5.2 Efficacy ranking rnalysis results

##### 3.5.2.1 Endometrial thickness

Using SUCRA to analyze the difference in the efficacy of three interventions to improve the endometrial thickness of PCOS patients, the results showed that the order of pregnancy rate from high to low was: Acupuncture & Moxibustion combined with Clomiphene (C), Acupuncture & Moxibustion (A), Clomiphene (B) (**Supplementary Figure 3**).

### 3.6 Subgroup analysis results

#### 3.6.1 Network relationship analysis results

##### 3.6.1.1 Effect of different acupuncture & moxibustion frequency on ovulation rate

**Figure 10:**
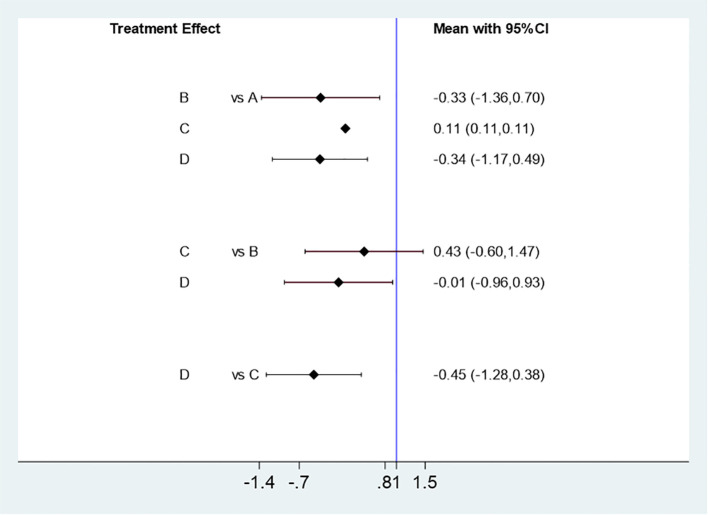
Mesh Meta Analysis Results of the Influence of Four Acupuncture & Moxibustion Frequencies on Ovulation Rate.

Six studies (5, 11–15) were effectively included to analyze the influence of different Acupuncture & Moxibustion treatment frequency on ovulation rate. Four kinds of Acupuncture & Moxibustion treatment frequency: once every other day, stop acupuncture after the follicle diameter is greater than or equal to 18 mm (A), and observe 98 cases. Treatment frequency: once every other day (B), observation and study of 40 cases. Treatment frequency: Once every other day, after the follicle diameter is greater than or equal to 18mm, once a day (C), 91 cases were observed and studied. Treatment frequency: Twice a week (D), 458 cases were observed and studied. The results of the mesh meta-analysis showed that A was compared to B [MD=0.41,95% CI (-1.89,2.70)], A was compared to C [MD=0.41,95% CI (-1.89,2.70)], A was compared to D [MD=0.41,95% CI (-1.89,2.70)], B was compared to C [MD=0.41,95% CI (-1.89,2.70)], B was compared to D [MD=0.41,95% CI (-1.89,2.70)], C was compared to D [MD=0.41,95% CI (-1.89,2.70)], P=0.2926, P>0.05, and the difference was not significant. It has statistical significance (**Figure 10**).

##### 3.6.1.2 Influence of different acupuncture & moxibustion frequency on pregnancy rate

**Figure 11:**
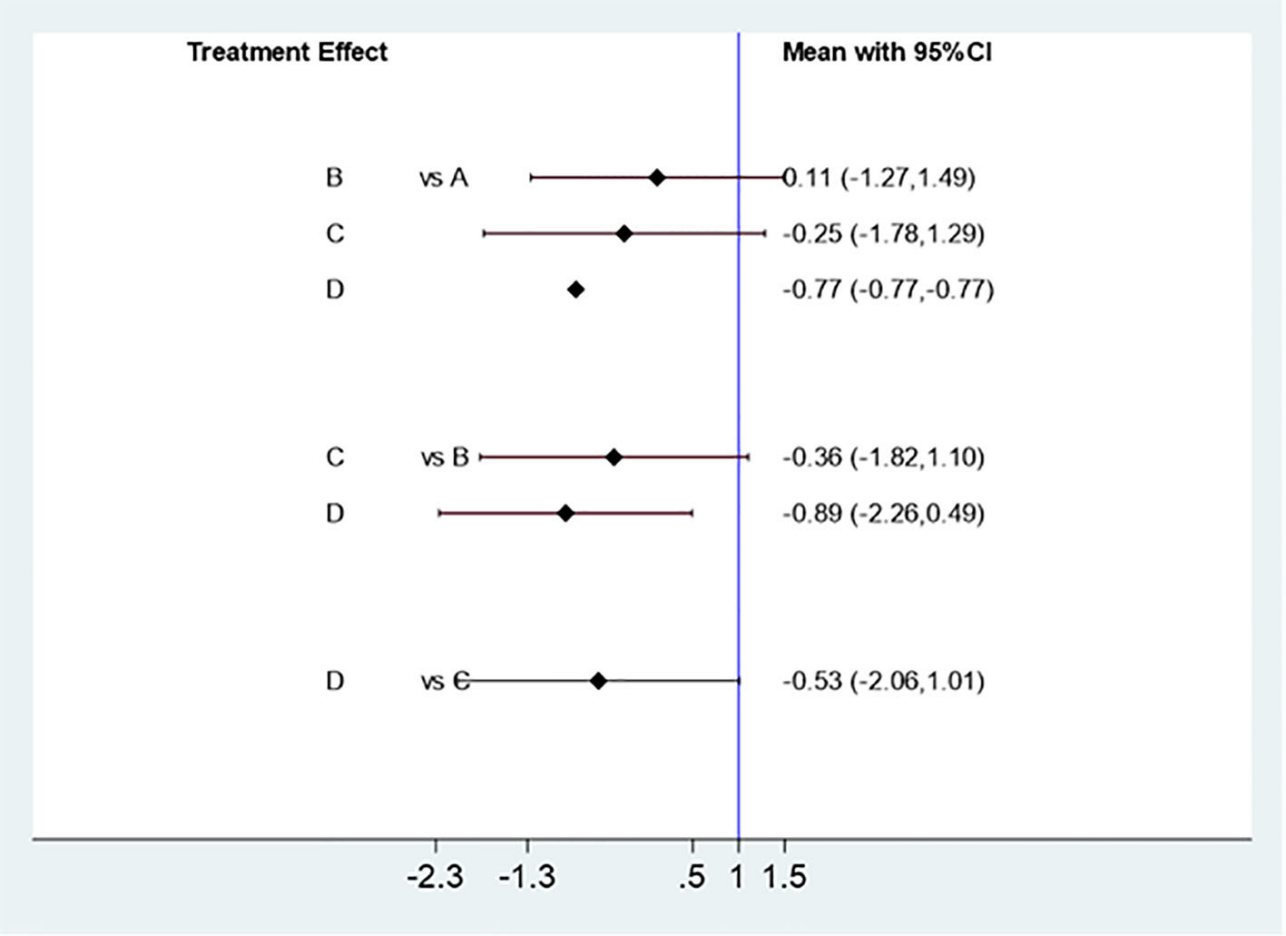
Mesh Meta Analysis Results of the Influence of Four Acupuncture & Moxibustion Frequencies on Pregnancy Rate.

Six studies (5, 11–15) were effectively included to analyze the influence of different Acupuncture & Moxibustion treatment frequency on pregnancy rate. 4 kinds of Acupuncture & Moxibustion treatment frequency: treatment frequency: once every other day, stop acupuncture (A) after the follicle diameter is greater than or equal to 18 mm, and observe 98 cases. Treatment frequency: Once every other day (B), observe 40 cases. Treatment frequency: Once every other day, after the follicle diameter is greater than or equal to 18mm, once a day (C), 91 cases were observed and studied. Treatment frequency: Twice a week (D), 458 cases were observed and studied. The results of the mesh meta-analysis showed that A was compared to B [MD=0.41,95% CI (-1.89,2.70)], A was compared to C [MD=0.41,95% CI (-1.89,2.70)], A was compared to D [MD=0.41,95% CI (-1.89,2.70)], B was compared to C [MD=0.41,95% CI (-1.89,2.70)], B was compared to D [MD=0.41,95% CI (-1.89,2.70)], C was compared to D [MD=0.41,95% CI (-1.89,2.70)], P=0.5002, P>0.05, and the difference was not significant. It has statistical significance (**Figure 11**).

##### 3.6.1.3 Effect of different acupuncture & moxibustion treatment methods on ovulation rate

**Figure 12:**
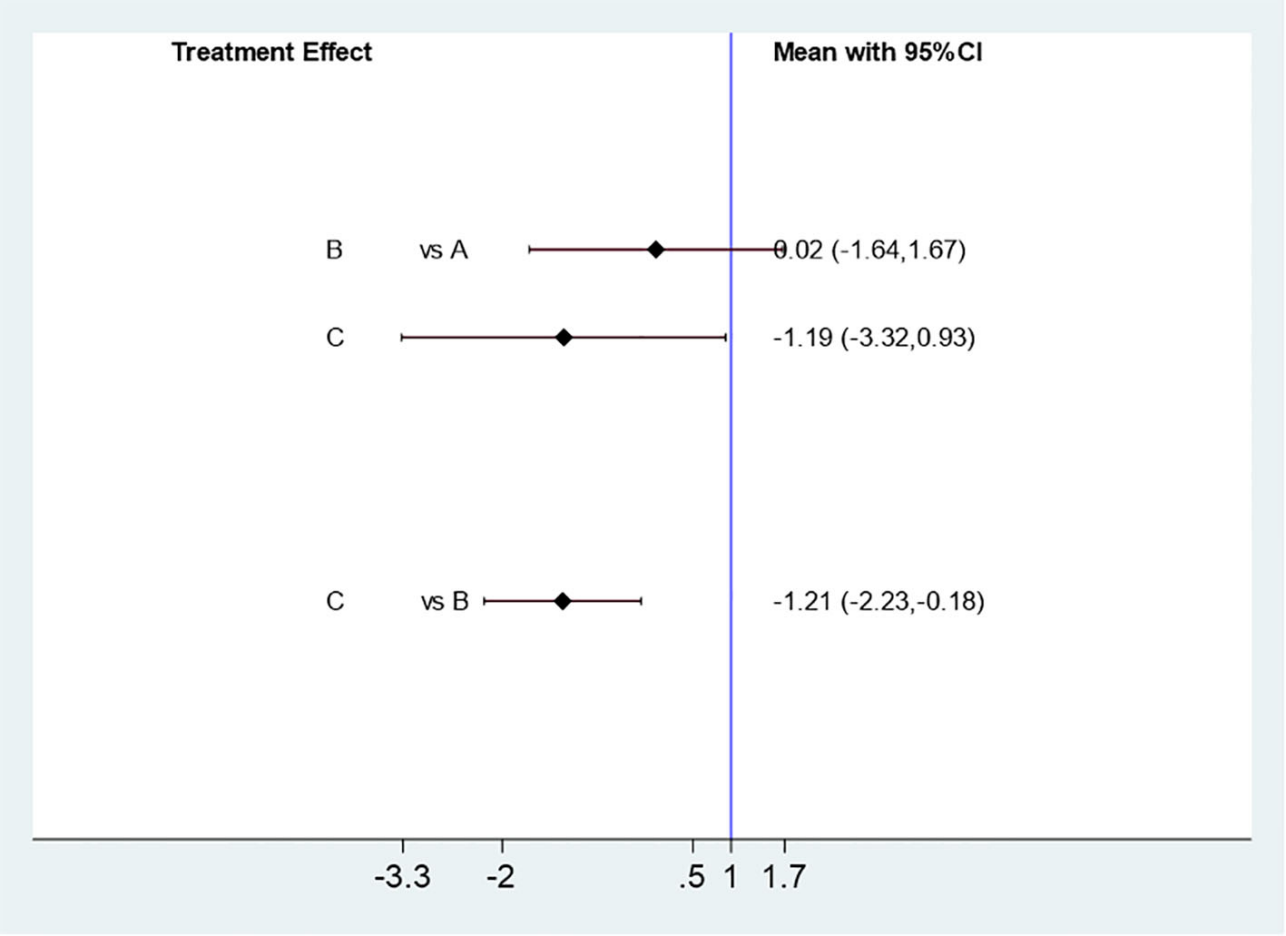
Mesh Meta Analysis Results of the Influence of Three Acupuncture & Moxibustion Methods on Ovulation Rate.

Five studies (11–15) were effectively included to analyze the effect of different Acupuncture & Moxibustion treatment methods combined with Clomiphene on ovulation rate. Three intervention measures: Acupuncture & Moxibustion combined with clomiphene (A) in 60 cases, Electroacupuncture combined with clomiphene (B) in 273 cases, and Warm Acupuncture & Moxibustion combined with Clomiphene (C) in 81 cases. The results of the mesh meta-analysis showed that there was a statistically significant difference between A and B [MD=0.02,95% CI (-1.64,1.67)], A and C [MD=-1.19,95% CI (-3.32,0.93)], and B and C [MD=-1.21,95% CI (-2.23, -0.18)], P=0.0210, P<0.05 (**Figure 12**).

##### 3.6.1.4 Influence of different acupuncture & moxibustion treatment methods on pregnancy rate

**Figure 13:**
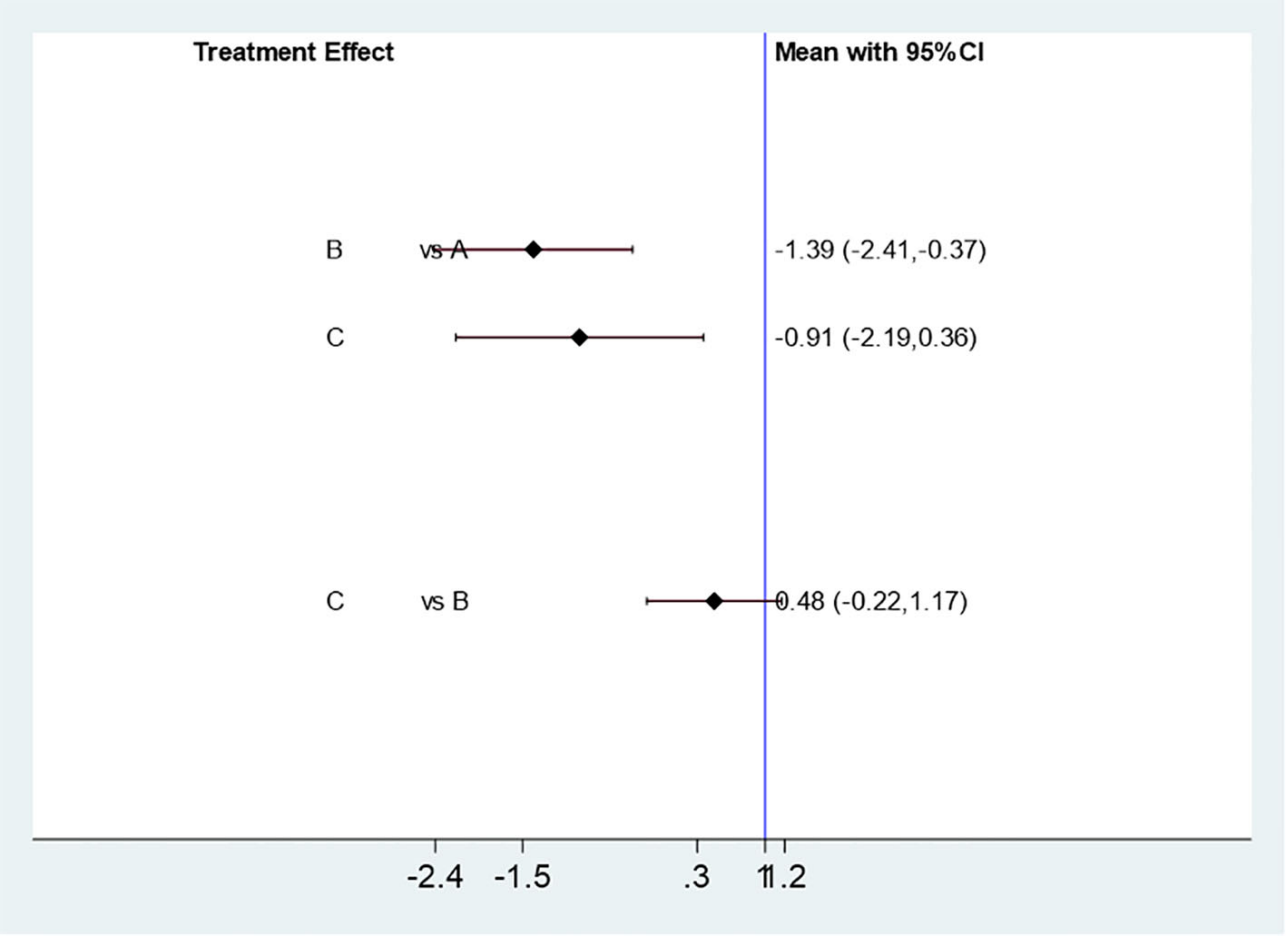
Mesh Meta Analysis Results of the Influence of Three Acupuncture & Moxibustion Methods on Pregnancy Rate.

Five studies (11–15) were effectively included to analyze the effect of different Acupuncture & Moxibustion treatment methods combined with Clomiphene on pregnancy rate. Three interventions: Acupuncture & Moxibustion combined with Clomiphene (A) in 60 cases, Electroacupuncture combined with Clomiphene (B) in 273 cases, and Warm Acupuncture & Moxibustion combined with clomiphene (C) in 79 cases. The results of the mesh meta-analysis showed that there was no statistically significant difference between A and B [MD=-1.39,95% CI (-2.41, -0.37)], A and C [MD=-0.91,95% CI (-2.19,0.36)], and B and C [MD=0.48,95% CI (-0.22,1.17)], P=0.1801, P>0.05 (See **Figure 13**).

#### 3.6.2 Efficacy ranking analysis results

##### 3.6.2.1 The difference of curative effect of four acupuncture & moxibustion treatment frequencies on ovulation rate

Using SUCRA to analyze the difference in the efficacy of four Acupuncture & Moxibustion methods to improve the ovulation rate of PCOS patients, the results showed that the order of pregnancy rate from high to low was: once every other day, after the follicle diameter was greater than or equal to 18 mm, once a day (C), once every other day (B), twice a week (D), once every other day, after the follicle diameter was greater than or equal to 18 mm, stop acupuncture (A) (**Supplementary Figure 4**).

##### 3.6.2.2 The difference of curative effect of four Acupuncture & Moxibustion treatment frequencies on pregnancy rate

Using SUCRA to analyze the difference in the efficacy of four Acupuncture & Moxibustion methods to improve the pregnancy rate of PCOS patients, the results showed that the order of pregnancy rate from high to low was: once every other day (B), once every other day, stop Acupuncture when the follicle diameter was greater than or equal to 18 mm (A), once every other day, once every other day when the follicle diameter was greater than or equal to 18 mm, once a day (C), twice a week (D) (**Supplementary Figure 5**).

##### 3.6.2.3 The difference of curative effect of three acupuncture & moxibustion methods on ovulation rate

Using SUCRA to analyze the difference in the efficacy of three Acupuncture & Moxibustion methods to improve the ovulation rate of PCOS patients, the results showed that the order of pregnancy rate from high to low was: electroacupuncture combined with clomiphene (B), Acupuncture & Moxibustion combined with clomiphene (A), warm Acupuncture & Moxibustion combined with clomiphene (C) **Supplementary Figure 6**).

##### 3.6.2.4 Influence of different acupuncture & moxibustion treatment methods on pregnancy rate

Using SUCRA to analyze the difference in the efficacy of three Acupuncture & Moxibustion methods to improve the pregnancy rate of PCOS patients, the results showed that the order of pregnancy rate from high to low was: Acupuncture & Moxibustion combined with Clomiphene (A), Warm Acupuncture & Moxibustion combined with Clomiphene (C), and Electroacupuncture combined with Clomiphene (B) (**Supplementary Figure 7**)

### 3.7 Adverse reactions

There are three studies to observe adverse reactions, including one study (12) to observe the occurrence of LUFS, 1.7% in the Acupuncture & Moxibustion combined with Clomiphene group (1 case/60 cases), 15% in the Clomiphene group (9 cases/60 cases), and to observe the occurrence of OHSS, 3.3% in the Acupuncture & Moxibustion combined with Clomiphene group (2 cases/60 cases), and 16.7% in the Clomiphene group (10 cases/60 cases); One study (11) observed the occurrence of adverse reactions such as nausea, vomiting, headache, and dermatitis, with a rate of 5% in the Electroacupuncture combined with Clomiphene group (2 cases/40 cases) and 25% in the clomiphene group (10 cases/40 cases); One study (15) observed the occurrence of diarrhea, with 5% in the Electroacupuncture combined with Clomiphene or Placebo group (25 cases/500 cases), 1.6% in the sham Acupuncture combined with Clomiphene or Placebo group (8 cases/500 cases), and 7.4% in the Electroacupuncture combined with Clomiphene or Placebo group (37 cases/500 cases), and 1.8% in the sham Acupuncture combined with Clomiphene or Placebo group (9 cases/500 cases).

## 4 Discussion

PCOS is a common endocrine and metabolic disorder in women of childbearing age, with complex and heterogeneous clinical manifestations. Studies have shown that the core causes and main endocrine features of PCOS are hyperandrogenism mia (HA) and insulin resistance (IR) (19). More than 60% of PCOS women can exhibit HA (20). Local high levels of androgens in the ovaries of PCOS patients hinder follicular development, resulting in anovulation or rare ovulation (21). Meanwhile, studies have shown that 50% to 70% of PCOS patients are troubled by IR (22), and IR can disrupt the hypothalamic pituitary ovarian axis, leading to ovulation disorders, rare menstruation, and even amenorrhea and infertility (23). The proportion of PCOS in patients with anovulatory infertility is as high as 75% (1). Therefore, how to promote follicular development and improve pregnancy rate in PCOS patients has become a focus and hotspot in the reproductive industry. At present, for the treatment of PCOS, the first step is to guide patients to improve their lifestyle, such as dietary intervention, exercise intervention, and behavioral intervention. It is recommended to carry out medication before and/or accompanied by medication (24). On this basis, for patients without improvement in treatment, ovulation promoting drugs are given (25). Clomiphene is widely used as a first-line ovulation promoting drug in patients with PCOS infertility. Although the use of clomiphene promotes ovulation rate, the pregnancy rate is low, and there are also side effects such as high miscarriage rate after conception, twin or multiple pregnancies, and ovarian hyperstimulation syndrome (26). Clomiphene is an artificially synthesized non-steroidal selective estrogen receptor modulator that can promote the secretion of LH and FSH, stimulate follicular growth, and induce ovulation. However, clomiphene has a certain anti estrogen effect and has a certain impact on the endometrium (27). Therefore, how to optimize the treatment plan and maintain the ovulation promoting effect of Clomiphene while improving the pregnancy rate of PCOS patients is an urgent problem to be solved in the reproductive industry.

Many studies have confirmed that Acupuncture & Moxibustion plays an active role in promoting follicular development (7, 8) and improving pregnancy outcomes (9, 10). Acupuncture & Moxibustion, as an alternative therapy, has gradually been used for PCOS treatment. In the previous meta-analysis and research, the clinical effect of Acupuncture & Moxibustion on PCOS was objectively evaluated by focusing on the impact of Acupuncture and Moxibustion on the pregnancy outcome of PCOS patients, as well as the impact on fasting insulin, blood sugar, blood lipids, sex hormones and other metabolic levels (28–30). This study focuses on the impact of Acupuncture & Moxibustion on endometrium, and evaluates whether Acupuncture & Moxibustion or Acupuncture & Moxibustion combined with Clomiphene has a synchronous effect on PCOS ovulation induction and endometrial development, so as to provide evidence-based medical evidence for optimizing the treatment plan and how to solve the problem of high ovulation rate and low pregnancy rate of Clomiphene. At the same time, based on the clinical effectiveness of Acupuncture & Moxibustion in treating PCOS, the subgroup analysis of this study further evaluates the impact of different Acupuncture & Moxibustion treatment methods and treatment frequency on outcome indicators, and collects evidence-based medical evidence to provide research direction for further optimizing Acupuncture & Moxibustion treatment methods.

The reticular meta-analysis showed that there was no statistical difference in the ovulation rate between groups among Acupuncture & Moxibustion, Clomiphene, Acupuncture & Moxibustion combined with Clomiphene, which was consistent with the clinical research results of Acupuncture & Moxibustion promoting follicular development. YULQ (31) found that Acupuncture combined with medicinal moxibustion, Warm Moxibustion box, and other methods can reduce the abnormally elevated LH, LH/FSH values and increase the abnormally decreased serum FSH levels in PCOS patients, maintain normal follicular growth, and promote normal ovulation in PCOS ovulation disorders. Its mechanism of action may be related to the regulation of Acupuncture & Moxibustion on neuroendocrine. ZHUHM’s research (32) found that Electroacupuncture can adjust the steady state of HPO axis in female rats and make the level of sex hormones in rats change regularly.

Mesh Meta analysis showed that Acupuncture & Moxibustion combined with Clomiphene has more significantly improvement of the pregnancy rate than just using Clomiphene or Acupuncture & Moxibustion. This provides important clinical research information on how to simultaneously improve ovulation and pregnancy rates in PCOS patients. However, there is a lack of research on PCOS live rate. At the same time, the reticular meta-analysis also showed that in the analysis of secondary indicators, Acupuncture & Moxibustion combined with Clomiphene could also significantly improve the endometrial thickness. This is consistent with the research of Lin Shuhuang et al. (33). A western medicine group, a physiotherapy group, and an Acupuncture & Moxibustion combined physiotherapy group (acupuncture at Sanyinjiao, uterus, Guanyuan, and Zhongji points on the eighth day of menstruation for three consecutive menstrual cycles) were set up to observe the effect of Acupuncture & Moxibustion combined with physiotherapy on endometrial receptivity of infertility patients with PCOS. Results the indexes of the Acupuncture & Moxibustion combined with physiotherapy group were better than those of the western medicine group and the physiotherapy group, with a statistically significant difference (P<0.5). From the perspective of mechanism research, Liu Xinyu et al. (34) found that Acupuncture at “Zusanli”, “Sanyinjiao”, and “Taichong” can enrich the expression of estrogen and progesterone receptors on the endometrium of mice with embryo implantation disorders, and synthesize sufficient estrogen and progesterone to exert physiological effects on target organs, stimulate endometrial proliferation and thickening, and create conditions for conception. However, there are few studies on the mechanism of Acupuncture & Moxibustion combined with Clomiphene. In the later stage, we should further study the mechanism of Acupuncture & Moxibustion combined with Clomiphene in the treatment of PCOS to provide a research basis for exploring the effect target.

Subgroup reticular meta-analysis showed that the influence of different Acupuncture & Moxibustion frequency on ovulation rate and pregnancy rate was not statistically significant. In the comparison of different Acupuncture & Moxibustion methods, it was found that the effect on the pregnancy rate was not statistically significant, but compared with Acupuncture & Moxibustion combined with Clomiphene, Warm Acupuncture & Moxibustion combined with Clomiphene, Electroacupuncture combined with Clomiphene could significantly improve the pregnancy rate, and the difference was statistically significant. There are few studies on the influence of Acupuncture & Moxibustion frequency or Acupuncture & Moxibustion treatment methods on the efficacy of PCOS. Therefore, the number of literature included in this study is limited, the number of research samples is relatively insufficient, and the results obtained are not reliable. We hope to increase the sample size for further research in the future.

For the analysis of adverse reactions, although only three studies were included, Acupuncture & Moxibustion combined with Clomiphene can reduce the incidence of LUFS and OHSS compared with the use of clomiphene alone, which is consistent with the analysis results of XJ. Liu (35) and Fu HY (36). At the same time, the combination of Acupuncture & Moxibustion and Clomiphene can reduce the occurrence of physical adverse reactions such as nausea, vomiting, headache and dermatitis compared with the use of clomiphene alone. It shows that the combination of acupuncture & moxibustion can reduce the adverse reactions of Clomiphene alone. Regarding the incidence of diarrhea and bruising reported in the included article, the combination of Electroacupuncture with Clomiphene or Placebo group is higher than the sham Acupuncture combined with Clomiphene or Placebo group, which may be related to the intervention methods of the two groups. More research is needed to expand the sample size for in-depth analysis.

Adiponectin(APN) is closely related to the core causes of PCOS, including hyperandrogenic mia (HA) and insulin resistance (IR), and is a key adipokine involved in regulating various reproductive activities (37). APN is a commonly used indicator for evaluating the disease progression and predicting long-term complications in patients with PCOS (38, 39). The included study did not involve the observation of this indicator, but Zhen Dou (40) found that Acupuncture at the eight points around the umbilicus can raise the pregnancy rate in obese type PCOS patients, which may be related to its effects in up-regulating serum APN and lowering BMI, WHR, HOMA-IR, and serum LEP levels. Liqing Yu (41) found that Acupuncture combined with medicine is better than just electroacupuncture for obese PCOS patients by improving obesity-related indexes, insulin sensitivity, and APN level. This indicates that acupuncture-medicine therapy is worth clinical popularization. There are few similar studies, and there is also a lack of research on the mechanism of Acupuncture & Moxibustion regulating adiponectin in the treatment of PCOS. In the later stage, more in-depth clinical and related mechanism studies can be carried out from the perspective of Acupuncture & Moxibustion regulating adiponectin, providing more detailed evidence-based medical evidence for Acupuncture & Moxibustion treatment.

As mentioned above, Acupuncture & Moxibustion is effective in improving the ovulation promoting effect and pregnancy outcome of PCOS patients. Among them, Acupuncture & Moxibustion combined with Clomiphene has more advantages in improving the pregnancy rate of PCOS patients. While making up for the high ovulation rate and low pregnancy rate of Clomiphene, it can also reduce the adverse reactions of the simple use of Clomiphene. In view of the shortcomings mentioned above in the current research, it is suggested that more high-quality RCT research should be carried out in the future, and pay attention to the influence of Acupuncture & Moxibustion treatment on PCOS live rate, and future research should also focus on the optimization of Acupuncture & Moxibustion programs to obtain the best cost-effective treatment measures.

## Data availability statement

The original contributions presented in the study are included in the article/**Supplementary Material**. Further inquiries can be directed to the corresponding author.

## Author contributions

LY: Writing – original draft, Writing – review & editing, Data curation, Supervision. WY: Data curation, Software, Writing – original draft. MS: Data curation, Writing – original draft. LL: Software, Writing – original draft. HL: Data curation, Supervision, Writing – original draft. RM: Data curation, Supervision, Writing – original draft. LP: Data curation, Supervision, Writing – original draft. YC: Software, Writing – original draft. KZ: Writing – review & editing.

## References

[1] Teede HJ Misso ML Costello MF Dokras A Laven J Moran L . Recommendations from the international evidence-based guideline for the assessment and management of polycystic ovarysyndrome. Hum Reprod (2018) 33(9):1602–18. doi: 10.1093/humrep/dey256 PMC6112576 30052961

[2] Shang S . Therapeutic effect of aspirin combined with clomiphene and chorionic gonadotropin in the treatment of infertility. China Pract Med (2022) 17(20):127–9. doi: 10.14163/j.cnki.11-5547/r.2022.20.038

[3] Xia Z Yan Z Jiang S Hao X Zheng X . Efficacy of letrozole versus clomiphene in the treatment of polycystic ovary syndrome. Chin J Clin rational Drug Use (2021) 14(29):126–8. doi: 10.15887/j.cnki.13-1389/r.2021.29.050

[4] Niandi MA . Comparison of clinical effects of letrozole and clomiphene in the treatment of infertility with polycystic ovary syndrome. China Foreign Med Treat (2022) 41(06):82–5. doi: 10.16662/j.cnki.1674-0742.2022.06.082

[5] Zhuo Y Wu J Lin W . The “regulating conception-governor vessel”acupuncture method for infertility of polycystic ovarian syndrome. Chin Acupuncture&Moxibustion (2016) 36(012):1237–41. doi: 10.13703/j.0255-2930.2016.12.002 29231358

[6] Smith JF Eisenberg ML Millstein SG Nachtigall RD Shindel AW Wing H . The use of complementary and alternative fertility treatment in couples seeking fertility care: data from a prospective cohort in the United States. Fertil Steril (2010) 93(7):2169–74. doi: 10.1016/j.fertnstert.2010.02.054 PMC2860047 20338559

[7] Yang LJ Wu J Yang L Zhou T Li HR Miao RQ . Effect on follicular development and pregnancy outcome treated with acupuncture and moxibustion therapy of Tiaochongren Gushenyuan in patients with luteal phase defect. Zhongguo Zhen Jiu (2019) 39(9):927–31. doi: 10.13703.0255/j.2930-2019.09.004 31544378

[8] Zheng C Luo D Pan L Huang J Bo Z . Therapeutic effects on infertility of ovulation failure in the patients with kidney deficiency treated with abdominal acupuncture and periodic therapy of Chinese herbal medicine. Zhongguo Zhen Jiu (2019) 39(5):482–6. doi: 10.13703/j.0255-2930.2019.05.006 31099218

[9] Guven PG Cayir Y Borekci B . Effectiveness of acupuncture on pregnancy success rates for women undergoing in *vitro* fertilization: A randomized controlled trial. J Obstet Gynecol (2020) 59(2):282–6. doi: 10.1016/j.tjog.2020.01.018 32127151

[10] Wu J-M Ning Y Ye Y-Y Liu Y-L Tang M Hu S . Effects of acupuncture on endometrium and pregnancy outcomes in patients with polycystic ovarian syndrome undergoing in *vitro* fertilization-embryo transfer: A randomized clinical trial. Chin J Integr Med (2022) 28(8):736–42. doi: 10.1007/s11655-022-3498-z 35419725

[11] Yu L Cao L Xie J . Therapeutic effect on ovulation and reproduction promotion with acupuncture and clomiphene in polycystic ovary syndrome. Chin Acupuncture Moxibustion (2018) 38(3):6. doi: 10.13703/j.0255-2930.2018.03.009 29701043

[12] Wang JX Li J Shen DW . Effects of acupuncture combined with cloid for treating womenwith polycystic ovary syndrome. Chinese Journal Of Faily Planning. Chin J Faily Plann 2022(005):030. doi: 3969/j.issn.1004-8189.2022.05.012

[13] Xu CX Mu YY . Efficacy analysis of Governor vessel warming needle moxibustion combined with clomiphene citrate in the treatment of infertility caused by polycystic ovary syndrome China Journal of Traditional Chinese Medicine and Pharmacy. China J Traditional Chin Med Pharm (2020) 35(4):4.

[14] Xue L Miao Y . Clinical effect of warm-needling moxibustion combined with clomiphene citrate for infertility with polycystic ovary syndrome and its effect on sex hormones and ovulation. J Of Chin Med 2022(054-010):164–7. doi: 10.13457/j.cnki.jncm.2022.10.038

[15] Wu XK Stener-Victorin E Kuang HY Ma HL Gao JS Xie LZ . Effect of acupuncture and clomiphene in Chinese women with polycystic ovary syndrome: A randomized clinical trial. JAMA (2017) 317(24):2502–14. doi: 10.1001/jama.2017.7217 PMC5815063 28655015

[16] Rotterdam ESHRE/ASRM-Sponsored PCOS consensus workshop group . Revised 2003 consensus on diagnostic criteria and long-term health risks related to polycystic ovary syndrome (PCOS). Fertil Steril (2004) 81(1):41–7. doi: 10.1093/humrep/deh098 14711538

[17] Endocrinology Group Society of Obstetrics and Gynecology Chinese Medical Association . Expert consensus on the diagnosis and treatment of polycystic ovary syndrome. Chin J Obstet Gynecol (2008) 43(7):553–5. doi: 10.3321/j.issn:0529-567x.2008.07.021

[18] Chinese Society of Obstetrics and Gynecology Endocrinology Group and guidelines expert group . Chinese diagnosis and treatment guide for polycystic ovary syndrome. Chin J Of Obstet Gynecol (2018) 53(1):2–6. doi: 10.3760/cma.j.issn.0529-567X.2018.01.002

[19] Wang J Wu D Guo H Li M . Hyperandrogenemia and insulin resistance: The chief culprit of polycystic ovary syndrome. Life Sci (2019) 236:116940. doi: 10.1016/j.lfs.2019.116940 31604107

[20] Livadas S Pappas C Karachalios A Marinakis E Tolia N Drakou M . Prevalence and impact of hyperandrogenemia in 1, 218 women with polycystic ovary syndrome. Endocrine (2014) 47(2):631–8. doi: 10.1007/s12020-014-0200-7 24752393

[21] Yang M Lin H-W Zhang H-Q Xu X-M Chen X . Androgen, androgen receptor and polycystic ovary syndrome. Acta ANATOMICA Sin (2018) 49(1):132–6. doi: 10.16098/j.issn.0529-1356.2018.01.023

[22] Catteau-Jonard S Dewailly D . Pathophysiology of polycystic ovary syndrome: the role of hyperandrogenism. Front Horm Res (2013) 40:22–7. doi: 10.1159/000341679 24002402

[23] Goodman Nf Cobin Rh Futterweit W Glueck JS Legro RS Carmina E . American association of clinical endocrinol ogists, American college of endocrinology, and androgen excess and PCOS society disease state clinical review: Guide to the best practices in the evaluation and treatment of Polycystic Ovary Syndrome - Part 2. Endocr Pract (2015) 21(12):1415–26. doi: 10.4158/EP15748.DSCPT2 26642102

[24] Eshre A . International evidence-based guideline for the assessment and management of polycystic ovary syndrome. (2018).

[25] Hang Hj Yy Y . Analysis on the curative effect of three different ovulation induction protocols in treatment of polycystic ovary syndrome. Maternal Child Health Care China (2018) 33(1):156–8.

[26] Nejad EST Abediasl Z Rashidi BH Nekoo EA Shariat M Amirchaghmaghi M . Comparisonof the efficacy of the aromatase inhibitor letrozole andclomiphen citrate gonadotropins in controlled ovarian hy-perstimulation: a prospective, simply randomized, clini-cal trial J. J Assist Reprod Genet (2008) 25(5):187–90. doi: 10.1007/s10815-008-9209-2 PMC2582070 18427974

[27] Yan J Yanhongchao . Efficacy of letrozole and clomiphene citrate for treating infertile women with polycystic ovarian syndrome. Chin J Fam Plann (2019) 27(8):1019–26. doi: 10.3969/j.issn.1004-8189.2019.08.010

[28] Zheng R Qing P Han M Song J Hu M Ma H . The effect of acupuncture on glucose metabolism and lipid profiles in patients with PCOS: A systematic review and meta-analysis of randomized controlled trials. Evid Based Complement Alternat Med (2021), 5555028. doi: 10.1155/2021/5555028 33824676 PMC8007365

[29] Li P Peng J Ding Z Zhou X Liang R . Effects of acupuncture combined with moxibustion on reproductive and metabolic outcomes in patients with polycystic ovary syndrome: A systematic review and meta-analysis. Evid Based Complement Alternat Med (2022), 3616036. doi: 10.1155/2022/3616036 35399633 PMC8991411

[30] Liao Z Fan H Fan H Chen X . Acupuncture for polycystic ovary syndrome: An overview of a protocol for systematic reviews and meta analyses. Med (Baltimore) (2021) 100(3):e24218. doi: 10.1097/MD.0000000000024218 PMC7837907 33546040

[31] Yu LQ Xie J Zhang FQ Shi Y Cao LY . Clinical study on acupuncture and moxibustion therapy for polycystic ovary syndrome (PCOS) with kidney deficiency and phlegm- dampness type. J ClinicalAcupuncture Moxibustion (2016) 32(2):10–5. doi: 10.19917/j.cnki.10050779.2016.02.003

[32] Zhu HM Nan S Suo CG Zhang QL Hu ML Chen R . Electro-acupuncture affects the activity of the hypothalamic-pituitary-ovary axis in female rats. Front Physiol (2019) 10(1):446. doi: 10.3389/fphys.2019.00466 31068836 PMC6491808

[33] Zhu XY Ji LP Liu CY Wu GH . Effect of acupuncture and moxibustion and Physiotherapy on PCOS Infertility Study on the influence of endometrial receptivity. HUNAN J OF TR ADITIONAL Chin Med (2015) 31(5):84–6. doi: 10.13935/j.cnki.sjzx.151227

[34] Liu XY Huang GY Zhang MM . Preliminary study on the mechanisms of acupuncture in promoting embryo implantation in rats. Chin J Integr Trad West Med (2007) 27(7):633–6. 17717926

[35] Liu XJ Shi WY Liu ZF Shi SQ Ke C Zhang PM . Effects of acupuncture on Luteinized Unruptured Follicle Syndrome: A meta-analysis of randomized controlled trials. Complement Therapies Med (2020) 49:102319. doi: 10.1016/j.ctim.2020.102319 32147029

[36] Fu HY Zhou HF Sun JH . Application of acupuncture and moxibustion and Moxibustion in Assisted Reproduction to Promote ovulation. Jilin J Traditional Chin Med (2013) 33(4):411–2.

[37] Singh A Choubey M Bora P Krishna A . Adiponectin and chemerin: contrary adipokines in regulating reproduction and metabolic disorders. Reprod Sci (2018) 25(10):1462–73. doi: 10.1177/1933719118770547 29669464

[38] Guo T Huang W Li G . Alteration of serum leptin adiponectin and resist in levels in PCOS patients treated by diane-35 alone or combined with metformin. J Pract Obstet Gynecology (2007) 23(11):669–72.

[39] Li X Li X Huang H-y Ma D Zhu M-W Lin J-F . Correlations between adipocytokines and insulin resistance in women with polycystic ovary syndrome. Zhonghua Yi Xue Za Zhi (2009) 89(37):2607–10. doi: 10.3760/cma.j.issn.0376-2491.2009.37.005 20137676

[40] Dou Z Ma S-H Song J-Y Xia T . Retrospective analysis on pregnancy outcomes and fat-related factors of treatment of endomorph PCOS infertility patients by acupuncture of 8 acupoints around umbilicus. Zhen Ci Yan Jiu (2021) 46(2):158–63. doi: 10.13702/j.1000-0607.200119 33788438

[41] Yu L Liao Y Wu H Zhao J Wu L Shi Y . Effects of electroacupuncture and Chinese kidney-nourishing medicine on polycystic ovary syndrome in obese patients. J Tradit Chin Med (2013) 33(3):287–93. doi: 10.1016/S0254-6272(13)60166-1 24024320

