# Supplementary material for: Meta analysis of ovulation induction effect and pregnancy outcome of acupuncture & moxibustion combined with clomiphene in patients with polycystic ovary syndrome
Source: Front Endocrinol (Lausanne). 2023 Nov 20;14:1261016. doi: 10.3389/fendo.2023.1261016 (PMC10698371; doi:10.3389/fendo.2023.1261016)
Supplement: Supplementary file 1 [file Table_1.doc]

Supplementary Table 1 inclusion of study characteristics table

| Study  No. | Study, pub-lication  year  (country) | No. of  patients  (O/A) | Age:  Mean  ±SD | Duration of infertility: mean  ±SD | Intervention | Control | Frequency of acupuncture  treatment  （One treatment time） | Period of treatment | Diagnostic  criteria | Side effects  and adverse  events | Type of out come | Summary of report outcome |
| --- | --- | --- | --- | --- | --- | --- | --- | --- | --- | --- | --- | --- |
| 1^[12]^ | Wang JX,  2022  (China) | I：60/60  C:  60/60 | I:30.6  ±3.2 C:31.5  ±3.2 | I:3.6  ±1.5  C:4.1  ±1.6 | Acupuncture & Moxibustion  + clomiphene | clomiphene | Once every 1day, continuous Acupuncture & Moxibustion treatment until ultrasound  the lower detection shows that the follicle diameter is ≥ 18mm.  (30min) | 3 months of continuous treatment | 2003 Rotterdam | Subcutaneous hematoma,  Gastrointestinal reactions. | Pregnancy,  Ovulation,  Number of mature follicles,  Mature follicle diameter, Endometrial condition, Unruptured follicles luteinization syndrome,  Ovarian overgrowth  stimulus syndrome. | After treatment, the total effective rate(93.3%) of the women in the observation group was significantly higher than that (80.0%) of the women in the control group. The rates of ovulation (86.7%) and clinical pregnancy(51.7%) of the women in the observation group were significantly higher than those (66.7% and 31.7%) of the women in the control group. The incidences of unruptured follicular luteinization syndrome (1.7%) and the ovarian hyperstimulation syndrome (3.3%) of the women in the observation group were significantly lower than those (15.0% and 16.7%) of the women in the control group(all P < 0.05),and the incidence of adverse reaction(3.3%) of the women in the observation group had no significant different from that (1.7%) of the in the control group(P >0.05) . |
| 2^[5]^ | Zhuo  YY  2016  (China) | I：50/50  C: 50/50 | I:29±5 C:28±5 | I:3.63  ±1.68 C:3.76  ±1.75 | Acupuncture | clomiphene | Treatment is given once every other day, followed by 4 treatments  B-ultrasound detection of follicles. Change the length of follicles tested to 18mm or above to  Acupuncture once a day until follicles are expelled and acupuncture treatment is stopped | 3 menstrual cycles | 2008  "Guidelines  for Diagnosis  and  Treatment of  Polycystic  Ovarian  Syndrome" in  China | NR | Pregnancy,  Ovulation,  Menstrual cycle, Endometrial condition,  Sex hormone level, Cervical mucus score,  BBT. | Result: After treatment, the ovulation rate in the observation group was 88.0% (44/50), which was better than 70.0% (35/50, P<0.05) in the control group; After treatment, the serum LH levels in both groups decreased compared to before treatment (both P<0.05, P<0.01), and the observation group was lower than the control group (P<0.05). Conclusion: Acupuncture & Moxibustion can promote the Menstrual cycle of PCOS patients to return to normal, increase the thickness of endometrium, promote the maturation of follicles, effectively reduce the level of serum LH, improve ovarian function, and effectively increase the ovulation rate, which is better than oral clomiphene. |
| 3^[15]^ | Wu XK  2017  (China) | I1：250/235  I2  250/223  C1:  250/236  C2:  250/232 | I1:28.2  ±3.4  I2: 27.8  ±3.2  C1:  27.8  ±3.4  C2: 28  ±3.3 | I1:6..2  ±1.9  I2: 6.3  ±2.0  C1: 6.2  ±2.0  C2: 6.1  ±2.3 | I1：Electroacupuncture + clomiphene  I2：  Electroacupuncture + Placebo | C1:  Sham Acupuncture  + clomiphene  C2:  Sham Acupuncture  + Placebo | acupuncture administered twice a week for 30 minutes per  treatment | 4 menstrual cycles | 2003 Rotterdam | Diarrhea,  Dysmenorrhea,  Bruising | PCOSQ,  ChiQOL,  SF-36  Pregnancy,  Ovulation,  Sex hormone level,  BMI,  Emotional Scale | Among Chinese women with polycystic ovary syndrome, the  use of acupuncture with or without clomiphene, compared with control acupuncture and  placebo, did not increase live births. This finding does not support acupuncture as an  infertility treatment in such women |
| 4^[11]^ | YU  LQ  2018  (China) | I：40/38  C: 40/37 | I:30±4 C:29±5 | I:3.6  ±2.6 C:3.4  ±2.3 | Electroacupuncture  + clomiphene | clomiphene | From the 5th day of menstruation or drug withdrawal bleeding, the patients were treated once every other day and three times a week, and the follicle diameter was ≥ 18mm from continuous Acupuncture & Moxibustion to vaginal Ultrasonic testing | 3 months of continuous treatment  or  3 menstrual cycles | 2003 Rotterdam | Nausea, vomiting  Vomiting, Headache, dermatitis | Pregnancy,  Ovulation,  Endometrial condition,  Sex hormone level. | Conclusion: The combination of electroacupuncture and clomiphene has a definite therapeutic effect on promoting ovulation and promoting pregnancy in polycystic ovary syndrome. The serum E: and P levels are significantly increased, the endometrium thickens, and the type A rate increases. Its therapeutic effect is significantly better than the use of clomiphene alone, and it is safe and tolerable for adverse reactions. The mechanism may be related to the improvement of estrogen and Progestogen levels and the enhancement of endometrial receptivity. |
| 5^[13]^ | Xu  CX  2020  (China) | I：41/41  C: 41/41 | I:27.73  ±5.29  C:28.16±4.39 | I:4.01  ±0.76  C:3.71  ±1.52 | warming needle moxibustion+ clomiphene | clomiphene | The Acupuncture & Moxibustion method is to treat once every other day from the fifth day of menstruation. After four times of treatment, when the follicle length is ≥ 18mm detected by B-ultrasound, Acupuncture & Moxibustion is given once a day.  (30min) | 3 menstrual cycles | 2003 Rotterdam | NR | Pregnancy,  Ovulation,  Ovarian volume,  Number of follicles,  Sex hormone level. | After treatment, the volume and number of follicles on both sides of the ovaries in the observation group decreased significantly compared to the control group (P<0.05); The ovulation number and pregnancy rate of the observation group were significantly higher than those of the control group (P<0.05). The Menstrual cycle of the observation group was significantly shorter than that of the control group (P<0.05), but there was no significant difference in the early abortion rate between the two groups; The improvement of sexual hormone levels in the observation group was significantly better than that in the control group (P<0.05); The treatment effect of the observation group was significantly better than that of the control group (P<0.05). Conclusion: Acupuncture & Moxibustion combined with clomiphene has a significant effect on infertility patients caused by PCOS, which is worthy of clinical application |
| 6^[14]^ | Xue  LF  2022  (China) | I：40/40  C: 40/40 | I:29.45  ±6.68  C:27.53±3.81 | I:5.13  ±1.15  C:5.19  ± 1.20 | Acupuncture & Moxibustio  + clomiphene | clomiphene | Start Acupuncture & Moxibustion on the second day after menstruation, once every other day.  (40min) | 3 menstrual cycles | 2018  "Guidelines  for Diagnosis  and  Treatment of  Polycystic | NR | Pregnancy,  Ovulation,  Sex hormone level,  TCM symptom score. | Result: After treatment, the total effective rate of the treatment group was 87.50%, while the control group was 67.50%. The difference between the two groups was statistically significant (P<0.05). After treatment, the serum LH and T levels in both groups decreased compared to before treatment (P<0.05), and the LH and T levels in the treatment group were on average lower than those in the control group (P<0.05); After treatment, the ovulation rate and pregnancy rate in the treatment group were 82.50% and 42.50% respectively, while those in the control group were 60% and 20% respectively. The difference between the two groups was statistically significant (P<0.05). Conclusion: Acupuncture & Moxibustion plus clomiphene is effective in the treatment of POS infertility, which can improve the clinical symptoms of patients, regulate the level of sex hormones, and increase the ovulation rate and pregnancy rate. |
