# Supplementary material for: Meta analysis of ovulation induction effect and pregnancy outcome of acupuncture & moxibustion combined with clomiphene in patients with polycystic ovary syndrome
Source: Front Endocrinol (Lausanne). 2023 Nov 20;14:1261016. doi: 10.3389/fendo.2023.1261016 (PMC10698371; doi:10.3389/fendo.2023.1261016)

Supplementary Figures

Supplementary Figure 1: Sorting results of ovulation rate and efficacy of three intervention methods

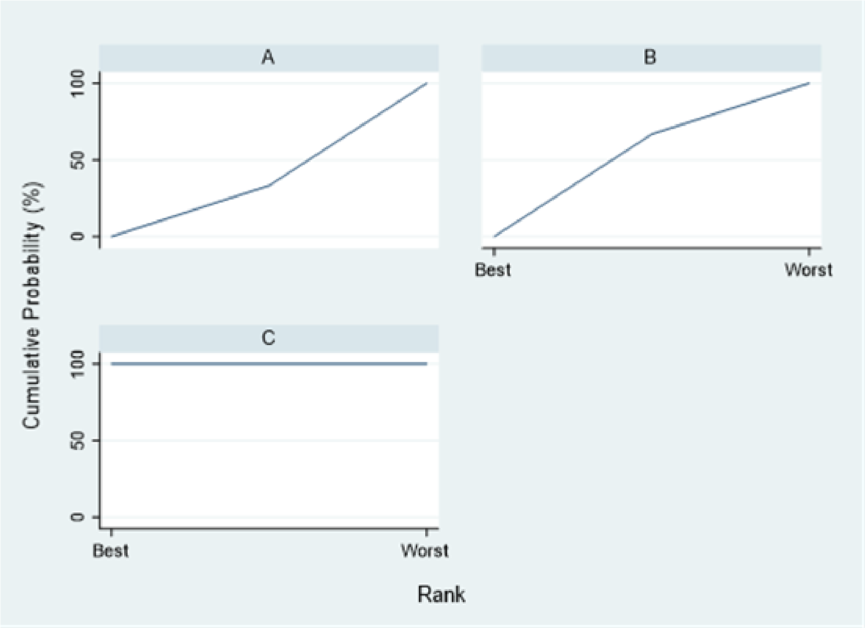

Supplementary Figure 2: sorting results of pregnancy rate and efficacy of three intervention methods

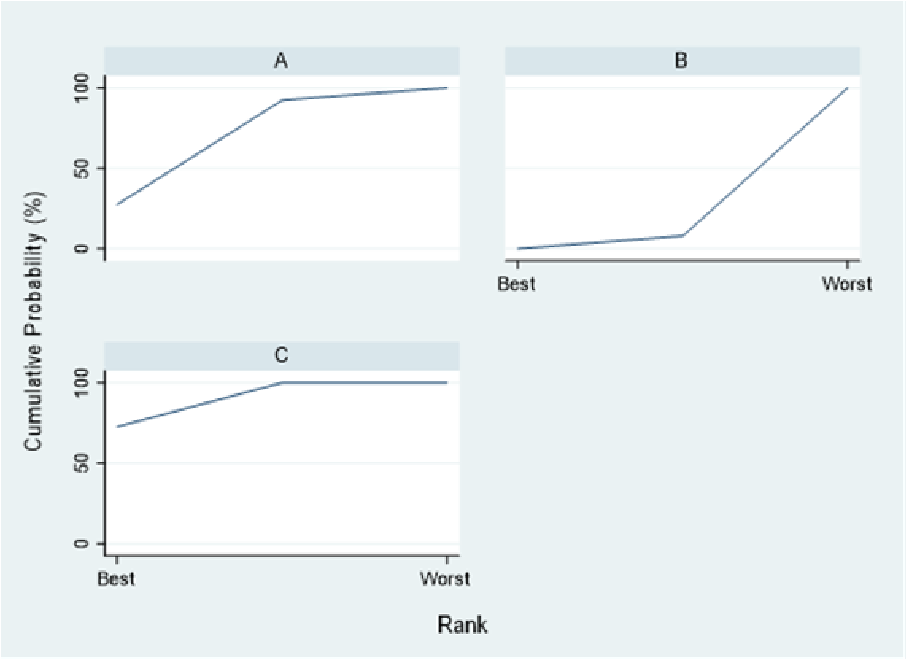

Supplementary Figure 3: ranking results of the efficacy of three intervention methods in improving endometrial thickness

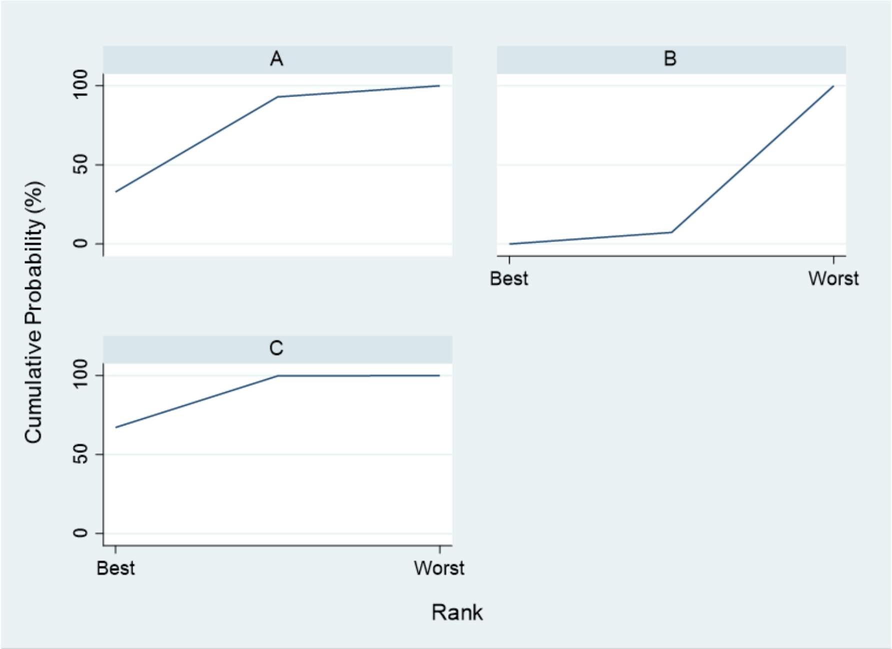

Supplementary Figure 4: Sorting Results of Effects of Four Acupuncture & Moxibustion Treatment Frequencies on Improving Ovulation Rate

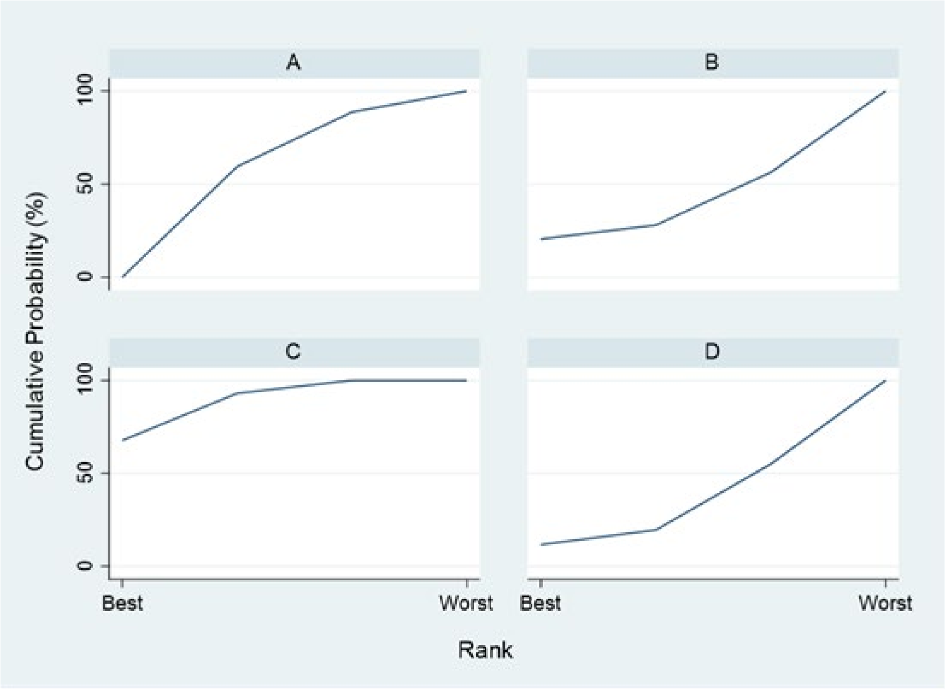

Supplementary Figure 5: Sorting Results of the Effects of Four Acupuncture & Moxibustion Treatment Frequencies on Improving Pregnancy Rate

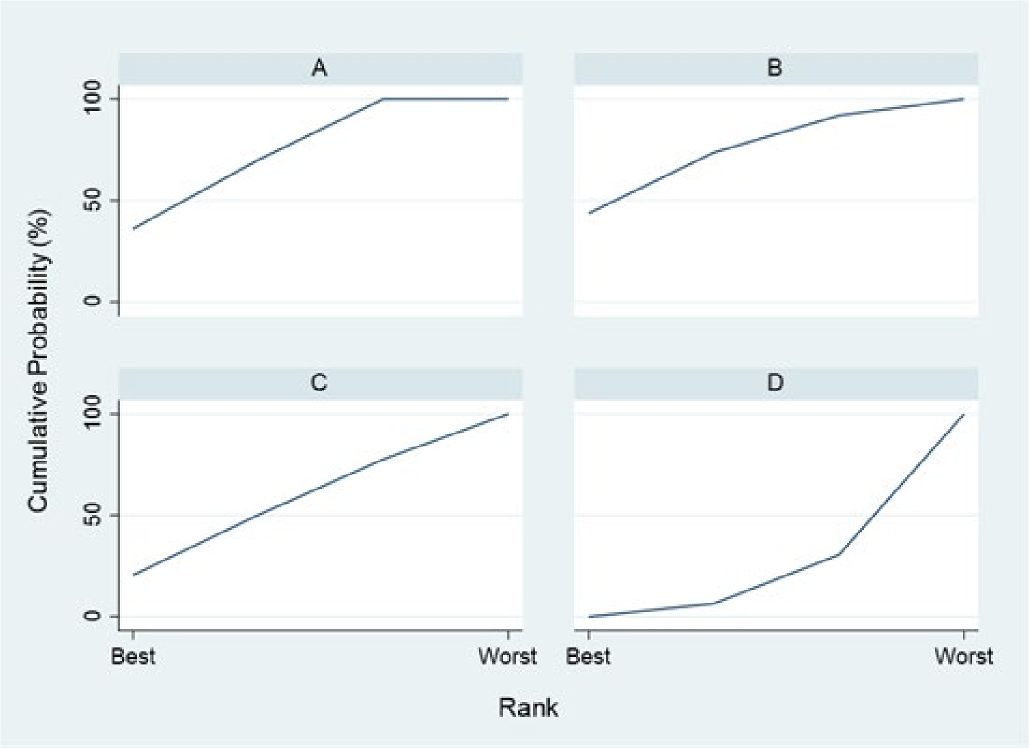

Supplementary Figure 6: Ranking Results of the Efficacy of three Intervention Methods in Improving Ovulation Rate

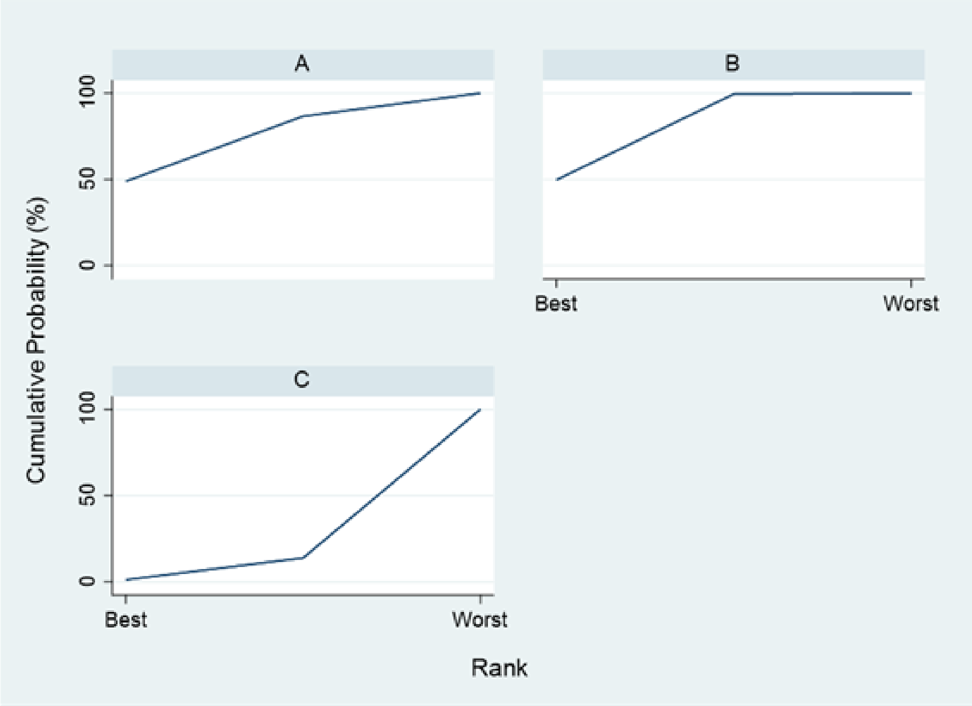

Supplementary Figure 7: Ranking Results of the Efficacy of three Intervention Methods in Improving Pregnancy Rate

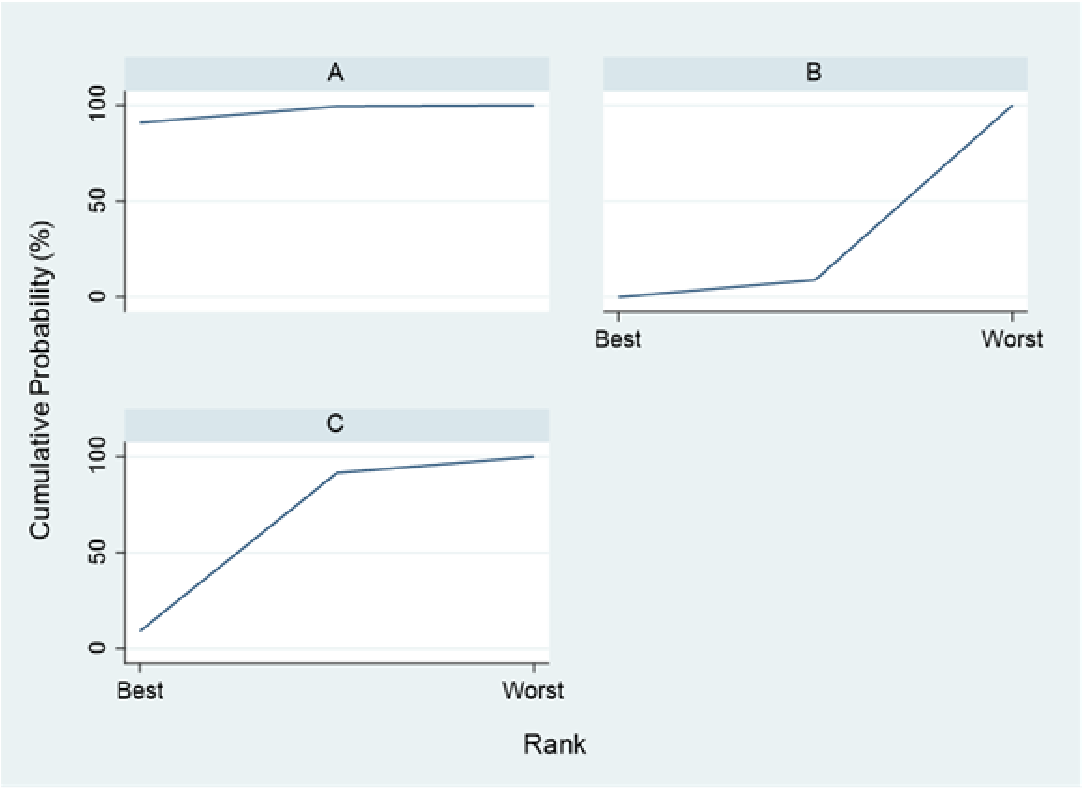

Supplement: Supplementary file 2 [file Image_1.pdf]
